# Connecting image inpainting with denoising in the homogeneous diffusion setting

**DOI:** 10.1186/s13662-025-03935-7

**Published:** 2025-03-28

**Authors:** Daniel Gaa, Vassillen Chizhov, Pascal Peter, Joachim Weickert, Robin Dirk Adam

**Affiliations:** Mathematical Image Analysis Group, Faculty of Mathematics and Computer Science, Saarland University, Campus E1.7, 66041 Saarbrücken, Germany

**Keywords:** 65D18, 68U10, 94A08, Diffusion, Denoising, Inpainting, Partial differential equations, Sampling

## Abstract

While local methods for image denoising and inpainting may use similar concepts, their connections have hardly been investigated so far. The goal of this work is to establish links between the two by focusing on the most foundational scenario on both sides – the homogeneous diffusion setting. To this end, we study a denoising by inpainting (DbI) framework. It averages multiple inpainting results from different noisy subsets. We derive equivalence results between DbI on shifted regular grids and homogeneous diffusion filtering in 1D via an explicit relation between the density and the diffusion time. We also provide an empirical extension to the 2D case. We present experiments that confirm our theory and suggest that it can also be generalized to diffusions with nonhomogeneous data or nonhomogeneous diffusivities. More generally, our work demonstrates that the hardly explored idea of data adaptivity deserves more attention – it can be as powerful as some popular models with operator adaptivity.

## Introduction

Investigating connections between different fields in image analysis has often been rewarded with deep structural insights. Consider, for example, the link between variational image inpainting [1–5] and optic flow computation [6–8] via the concept of the *filling-in effect*. This effect is due to the smoothness term (regularizer) of the models, which inserts information at locations where the data term is absent or small in magnitude. The gradient flow for minimizing the variational energy functional leads to partial differential equations (PDEs) with a diffusion term.

While the filling-in effect has an obvious benefit for image inpainting, it can also lead to more powerful optic flow methods. It produces a dense flow field from the sparse information of the data term. Surprisingly, the parts of the flow field that are filled in by the diffusion-like regularization terms are usually those with the highest confidence [9].

**Figure 1:**
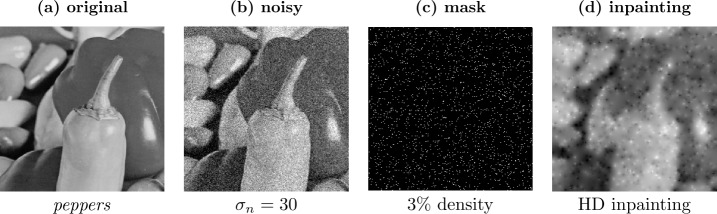
Homogeneous diffusion (HD) inpainting on the test image *peppers* ($256 \times 256$ pixels, image range $[0, 255]$) with additive Gaussian noise of standard deviation $\sigma _{n}=30$ that we do not clip. The mask pixels are randomly selected. Note that the inpainted pixels are more reliable, since they average noisy information from the neighborhood. The visual difference is also reflected by the mean squared error (MSE): The MSE of the noisy image in (b) is 904. Since the mask pixels are chosen randomly and are not changed by the inpainting, the MSE at mask pixel locations in (d) is still approximately 900. However, the total image MSE in (d) is only 475

Figure 1 shows a similar but hitherto hardly studied effect when performing *sparse inpainting* on noisy data. There the known data – the so-called *mask* – is a scattered set of pixels. The noisy mask pixels remain unchanged during the process, while the unknown areas in between are interpolated smoothly by averaging information from the noisy pixels. We thus again have a scenario, where *the filled-in data are more reliable than the known data*. In the present manuscript we study how far this idea can lead us.

### Our contribution

The goal of our work is to shed some light on the connections between PDE-based inpainting and denoising, two tasks which have coexisted for a long time, while their links have hardly been studied so far. We bridge this gap by a detailed investigation of the unconventional idea of denoising by inpainting. To facilitate a rigorous mathematical analysis, we focus on homogeneous diffusion. As will be explained below, it constitutes the most transparent and most foundational setting in both worlds.

The present paper builds upon our previous conference publication [10], in which the basic denoising by inpainting framework is established. This framework reconstructs a denoised version of an image by averaging the results of multiple inpaintings obtained from distinct masks. Furthermore, two concrete implementations of this framework are proposed in [10]: The first uses shifted regular masks and allows establishing a relation between denoising by inpainting and classical diffusion filtering in 1D, while the second uses probabilistic densification to adapt the masks to the image structures and enables an edge-preserving denoising behavior.

We extend the aforementioned results by a much broader study of the framework in [10], providing a fundamental understanding of the connections between PDE-based image inpainting and denoising. Since denoising methods can also be used as plug-and-play priors in algorithms for solving inverse problems [11–13], our relations between inpainting and denoising approaches may have an even broader application spectrum. Compared to [10], we introduce the following additional contributions:

- We show that the heuristically motivated DbI framework from [10] can be seen as a representative of a general probabilistic framework, for which we derive a sound theory. We argue that the denoising result obtained with such framework is an approximation of a minimum mean squared error (MMSE) estimate.
- We provide convergence estimates for the framework and propose a deterministic sampling approach to boost the convergence.
- We prove a general relation between the mask density of regular masks in the DbI framework and the diffusion time of homogeneous diffusion filtering in 1D. We also propose an empirical generalization of this result to 2D for uniform random masks.
- We integrate a step that optimizes the gray values at the selected mask pixels (tonal optimization) into the DbI framework. We investigate its effect on the MMSE estimate and perform experiments which confirm that tonal optimization can improve the denoising performance of DbI in practice.
- We show that the different spatial optimization approaches in the DbI framework correspond to specific posterior distributions. We compare two such strategies (that presented in [10] and a novel one) in terms of quality and provide the formulations for the respective probability distributions. Our experiments demonstrate that this data optimization leads to an edge-preserving denoising behavior.
- We replace homogeneous diffusion inpainting in the DbI framework by biharmonic inpainting and show that it is unable to improve denoising results. This confirms one of our key insights: The hitherto hardly practiced data optimization can be as powerful as widely used operator optimizations.

#### Why homogeneous diffusion?

Our decision to focus on homogeneous diffusion is based on several reasons:

- For denoising and image simplification, one should keep in mind that homogeneous diffusion filtering is equivalent to Gaussian convolution. The Gaussian is the only convolution kernel that is separable and rotation invariant. The diffusion evolution generates a Gaussian scale-space representation [14–16], which is one of the most widely-used scale-spaces and forms the basis of highly successful interest point detectors such as SIFT [17] and its numerous variants.
- In inpainting applications, homogeneous diffusion is particularly popular in inpainting-based compression [18], where one stores only a sparse subset of all pixels and reconstructs the image in the decoding phase by inpainting. By optimizing the stored data, homogeneous diffusion can achieve surprisingly faithful reconstructions [19]. Moreover, its simplicity allows a detailed theoretical analysis [20], it frees the user from specifying parameters, and one can achieve real-time performance on current PC hardware even for large images [21].
- Last but not least, there exist already well-understood connections between diffusion processes for denoising and other approaches, such as variational regularization methods [22, 23] and wavelets [24, 25], but also deep neural network architectures [26, 27]. Thus, establishing also connections to inpainting ideas gives more comprehensive insights into various paradigms beyond diffusion-based denoising.

This discussion also implies that *it is not the goal of the present paper to design novel approaches that outperform the most recent state-of-the-art approaches for denoising or inpainting.* This is reserved for future research that may benefit from the foundational insights in the our manuscript.

### Related work

Since we consider image inpainting as well as image denoising, we give an overview of some relevant methods from both fields and relate them to our work.

#### PDE-based denoising and inpainting

We borrow several ideas from sparse PDE-based inpainting methods [18]. We mostly restrict ourselves to homogeneous diffusion inpainting [28], which can be implemented very efficiently [21, 29–33], and – in spite of its simplicity – can produce convincing results for suitably chosen data [20, 34–40]. Especially on piecewise constant images, such as cartoon images, depth maps or flow fields, homogeneous diffusion inpainting in conjunction with edge or segment information performs very well [28, 33, 41–45]. This even allows some of these methods [43, 44] to outperform HEVC [46] on such data. Nonlinear diffusion inpainting methods, e.g., edge-enhancing diffusion (EED) inpainting [18, 47], can improve reconstruction quality for sparse inpainting, enabling lossy image codecs [18, 48, 49] competitive to JPEG [50] and JPEG2000 [51]. On the other hand, such methods are more complex due to their nonlinearity. This complexity also carries over to the data optimization process. Higher-order inpainting operators can also be used for sparse inpainting [18, 36, 49, 52, 53], but can be more sensitive to noise. The quality of PDE-based sparse inpainting approaches strongly depends on the stored data, and in our denoising by inpainting framework we incorporate ideas from spatial optimization [20, 30, 33, 34, 36–40] and tonal optimization [30, 36, 38, 39, 54]. To interpret the filtering results of the denoising by inpainting framework, we compare to classical diffusion-based image denoising methods. Aside from the simple homogeneous diffusion [14], we also consider methods that adapt the diffusion operator to the given image, namely linear space-variant diffusion [55] and nonlinear diffusion [56]. We choose these methods because they are closest conceptually so we expect them to provide useful insights.

#### Patch-based denoising and inpainting

Patch- or exemplar-based methods are another class of inpainting methods, and work especially well with textured data. The idea is to copy similar patches from known to unknown regions. Efros and Leung have proposed the first exemplar-based inpainting method [2], but many versions have been developed since then (e.g., [57–60]), including the method of Facciolo et al. for sparse inpainting [61]. Inpainting approaches combining PDE- and patch-based methods have also been presented [62, 63]. Inspired by the method of Efros and Leung [2], a patch-based denoising method called NL-means [64] has been proposed. It denoises an image based on a nonlocal weighted averaging of similar image patches. Other algorithms such as the famous BM3D algorithm [65] are also based on the filtering of image patches. These observations further substantiate the ties between denoising and inpainting. The NL-means method can even be interpreted as a case of a denoising by inpainting approach, although it does not use the inpainting ideas as directly as we do. Of course, a direct application of patch-based inpainting techniques would lead to the copying of erroneous noisy data, and not to a denoising effect.

#### Sparse signal approximation

A popular approach in the field of image denoising relies on the idea that signals (and images) can be represented as a linear combination of a smaller number of basis signals – so-called atoms – that are selected from a dictionary [66]. Such a dictionary might for example consist of the basis vectors of a suitable transform, that makes the signal representation sparse (e.g., a wavelet transform [67] or a discrete cosine transform (DCT) [68]). The task is to then find those atoms, that best represent the given signal [69–71]. To fill in missing information in images, several authors also consider sparse representations in some transform domain such as the DCT [72] or the shearlet domain [73]. This shows another bridge between the two tasks of denoising and inpainting. Hoffmann et al. [31] relate linear PDE-based inpainting methods to concepts from sparse signal approximation. They solve the inpainting problem with the help of discrete Green’s functions [74, 75], which can be interpreted as atoms in a dictionary. This allows for a sparse representation of the inpainting solution. Kalmoun et al. [32] follow a similar approach by solving homogeneous diffusion inpainting with the charge simulation method [76, 77]. An application of homogeneous diffusion inpainting with Green’s functions is the video codec by Andris et al. [29]. We justify certain design choices within the DbI framework with results from this field. Notably, homogeneous diffusion inpainting is based on the idea that the Laplacian of the reconstructed image is mostly sparse. On the other hand, the DbI framework combines multiple noisy sparse representations in order to get a denoised but nonsparse representation. The latter can be studied rigorously from a Bayesian denoising perspective, which is why we discuss this next.

#### Bayesian denoising

The study of denoising has also been carried out from a probabilistic perspective. Here, the assumption is that some prior information regarding the noise distribution and/or the image distribution is available. This can be incorporated in a denoising framework through Bayes’ rule, such that the final denoised result is conditioned on this information about the distributions. The latter provides a correspondence between classical denoising variational methods and specific Bayesian priors [78–80]. The standard approach is to employ statistical inference approaches, such as maximum likelihood (ML) estimation, maximum a posteriori (MAP) estimation, or minimum mean squared error (MMSE) estimation. Both the MAP and MMSE approach rely on a posteriori density, and as such they require a model of the distribution of considered classes of images. One of the first such models uses a Gibbs distribution for the prior [81]. Subsequently, a number of works have built upon this idea. The most relevant to our setting is that by Larsson and Selen [82], which studies MMSE estimation in the context of sparse vector representations. Our sparse inpaintings can be interpreted as such sparse vector representations. Moreover, in the current work we show that the averaging performed in [10] is, in fact, a Monte Carlo approach to approximate an MMSE estimate.

#### Cross-validation

We also see the work of Craven and Wahba [83] on (generalized) cross-validation as conceptually related to parts of our work. Cross-validation can be used to optimize parameters in denoising models [82–84]. It removes data points from given noisy observations and judges the quality of a parameter selection in terms of the model’s capability to reconstruct the data at these locations. Related ideas are also pursued in [85]. Probabilistic densification [42] and sparsification [39], two concepts from spatial optimization that we consider in our framework, also use the error of the inpainted reconstruction at left out locations – in our case also on noisy data. Yet, both applications differ, as the goal of the latter methods is to construct an inpainting mask and not to optimize model parameters.

#### Neural denoising and inpainting

In recent years, many very powerful methods for inpainting and denoising have been proposed that rely on neural networks. They are, however, not a topic of our paper, since we aim at gaining structural insights into the connections between inpainting and denoising. Such results on classical approaches are still relevant in the learning era [78]. They may serve as foundations for deep learning-based methods, and model- and learning-based approaches may be fused to obtain powerful and transparent algorithms. It is our hope that in the long run, our insights can also be beneficial to neural approaches.

### Paper organization

In Sect. 2 we briefly introduce the basic idea behind diffusion filtering and its application to image denoising and image inpainting. In Sect. 3 we present the framework for denoising by inpainting from [10] and show that it can be interpreted as a Monte Carlo approach for approximating an MMSE estimate. We additionally provide convergence results, and suggest a method to boost the convergence by employing low-discrepancy sequences. In Sect. 4 we relate denoising by inpainting with nonadaptive masks to classical diffusion filtering. In Sect. 5 we present strategies for adaptively selecting the mask pixels in the DbI framework, which leads to space-variant denoising behavior. Our experiments and results are presented in Sect. 6, and we conclude the paper in Sect. 7.

## Basics of diffusion filtering

In its original context of physics, diffusion is a process that equilibrates particle concentrations. When working with images, we interpret the gray values as particle concentrations and use diffusion processes as smoothing filters that balance gray value differences. To this end, we define the original grayscale image as a function $f:\Omega \to \mathbb{R}$, with $\Omega \subset \mathbb{R}^{2}$ being a rectangular image domain. Similarly, $u:\Omega \times [0,\infty ) \to \mathbb{R}$ denotes the evolving, filtered image. Then the diffusion evolution is described by the following PDE:

1 $$ \partial _{t} u(\boldsymbol{x}, t) = \operatorname{div}(g \boldsymbol{\nabla} u( \boldsymbol{x}, t)) \quad \mbox{for }\boldsymbol{x} \in \Omega , \,\,t \in (0, \infty ). $$

Here *t* denotes time, $\boldsymbol {\nabla}=(\partial _{x}, \partial _{y})^{\mathsf{T}}$ is the spatial gradient, $\operatorname{div}(\boldsymbol{v}) = \partial _{x} v_{x} + \partial _{y} v_{y}$ is the spatial divergence, and the scalar diffusivity *g* determines the local smoothing activity. We discuss different choices of *g* in Sect. 2.1. Note that *g* can be extended to a diffusion tensor to introduce anisotropy into the process [86], but since we do not consider such a case in this paper, we refrain from discussing it here. We equip the PDE with an initial condition at time $t=0$ and reflecting boundary conditions at the image boundary *∂*Ω:

2 u(x,0)=f(x)for x∈Ω,

3 $$\partial_{n}u(x,t)=0\;\text{for }x\in \partial \Omega ,\;\;t\in (0,\infty ),$$

where ***n*** is the outer normal vector at the image boundary. Solving this initial boundary value problem for *u* yields a family of filtered images $\{u(\cdot , t) \mid t \geq 0\}$.

### Diffusion for image denoising

In image denoising the image *f* is a noisy version of the noise-free ground truth image $f_{r}$. In our case we assume zero-mean additive white Gaussian noise, i.e., $f = f_{r} + n$ with $n\sim \mathcal{N}(0, \sigma _{n}^{2})$. Diffusion processes are good candidates for image denoising tasks thanks to their smoothing properties. Depending on the form of the diffusivity *g*, different processes are obtained.

#### Homogeneous diffusion

By setting $g \equiv 1$, (1) simplifies to $\partial _{t} u = \Delta u$, with $\Delta u = \partial _{xx} u + \partial _{yy} u$ being the Laplacian operator. The resulting process is known as *homogeneous diffusion* [14]. Its analytical solution in the unbounded image domain $\mathbb{R}^{2}$ is given by a convolution of the original image with a Gaussian kernel $K_{\sigma}$ with standard deviation $\sigma = \sqrt{2t}$. The resulting images $\{u(\cdot , t) \mid t \geq 0\}$ constitute the so-called Gaussian scale-space [14, 87]. Since *g* is selected to be constant, the smoothing strength is the same across the entire image. Therefore, not only the noise is reduced, but also semantically important image structures such as edges are smoothed.

#### Linear space-variant diffusion

To overcome the drawbacks of homogeneous diffusion, one can make the process space-variant by selecting a diffusivity function that varies depending on the structure of the *initial* image *f* [55]. This is called *linear space-variant diffusion*. If edges and other high-gradient features are to be preserved, the diffusivity should be decreasing with increasing gradient magnitude of the image, so that that the smoothing would be reduced at edges. An example for a suitable function is the Charbonnier diffusivity [88],

4 $$ g(|\boldsymbol{\nabla} f|^{2}) = \frac{1}{\sqrt{1+\frac{|\boldsymbol{\nabla} f|^{2}}{\lambda ^{2}}}}, $$

where $| \cdot |$ denotes the Euclidean norm. The contrast parameter $\lambda > 0$ is used to distinguish locations where smoothing should be applied (for $|\boldsymbol{\nabla} f| \ll \lambda $, we get $g_{\lambda} \to 1$) and locations where it should be reduced (for $|\boldsymbol{\nabla} f| \gg \lambda $, we obtain $g_{\lambda} \to 0$).

#### Nonlinear diffusion

Alternatively, one can make the diffusivity function *g* dependent on the *evolving* image *u*. This allows updating the locations where smoothing is reduced during the evolution, by choosing them based on the image *u*, which becomes gradually smoother and less noisy. The resulting process $\partial _{t} u = \operatorname{div}(g(| \boldsymbol{\nabla} u |^{2}) \boldsymbol{\nabla} u)$ is *nonlinear* [56]. The feedback mechanism throughout the evolution helps steering the process to achieve better results.

### Diffusion for image inpainting

Diffusion processes can also be used to fill in missing information in images [28, 47, 89]. Particularly, they allow reconstructing an image from only a small number of pixels by propagating information from known to unknown areas [18]. The set of known pixels is called the *inpainting mask* and is denoted by $K \subset \Omega $. To recover the image, the information at the unknown locations is computed as the steady state ($t \to \infty $) of a diffusion process, while the values at mask locations are preserved. The parabolic inpainting formulation is obtained by modifying (1) and (2) accordingly:

5 $$\partial_{t}u(x,t)=\mathrm{div}(g\nabla u(x,t))\;\text{for }x\in \Omega \setminus K,\;\;t\in (0,\infty ),$$

6 u(x,t)=f(x)for x∈K,t∈[0,∞),

7 u(x,0)=0for x∈Ω∖K,

8 $$\partial_{n}u(x,t)=0\;\text{for }x\in \partial \Omega ,\;\;t\in (0,\infty ).$$

For $g\equiv 1$, (5) is the homogeneous diffusion PDE [14] and we talk about *homogeneous diffusion inpainting* (also called *harmonic inpainting*). We almost exclusively consider homogeneous diffusion inpainting in the remainder of this paper, so we set $g\equiv 1$ in the following. Instead of computing the steady state of the parabolic diffusion equation, we may solve the corresponding boundary value problem:

9 −Δu(x)=0for x∈Ω∖K,

10 u(x)=f(x)for x∈K,

11 $$\partial_{n}u(x)=0\;\text{for }x\in \partial \Omega .$$

The problem may be written equivalently using the variational formulation

12 $$ \min _{u}\int _{\Omega}|\nabla u(\boldsymbol{x})|^{2}\,d\boldsymbol{x}, \text{ such that } u(\boldsymbol{x}) = f(\boldsymbol{x}) \text{ for } \boldsymbol{x} \in K. $$

This suggests the interpretation that the inpainting is designed to penalize the gradient magnitude of the reconstruction, i.e., it inherently promotes smoothness. In order to simplify the discretization of the boundary value problem formulation, we introduce a mask indicator function $c=1_{K}$ (we use the term *mask* synonymously for the set *K* and the function *c*), that takes the value 1 at points from *K* and 0 elsewhere. This allows us to combine (9) and (10) into a single equation

13 $$ \bigl(c(\boldsymbol{x}) + (1 - c(\boldsymbol{x})) (-\Delta )\bigr)u(\boldsymbol{x}) = c( \boldsymbol{x})f(\boldsymbol{x}) \quad \text{for } \boldsymbol{x} \in \Omega . $$

### Discrete homogeneous diffusion inpainting

Since we are working with digital images, the above considerations need to be translated to the discrete setting. We therefore discretize the images on a regular pixel grid of size $n_{x} \times n_{y}$. Then we write them as vectors of length $N = n_{x} n_{y}$ that are obtained by stacking the discrete images column-by-column, e.g., $\boldsymbol{f}, \boldsymbol{u} \in \mathbb{R}^{N}$. Furthermore, let $\boldsymbol{L}\in \mathbb{R}^{N\times N}$ denote the five-point stencil discretization matrix of the negated Laplacian $(-\Delta )$ with reflecting boundary conditions $\partial _{\boldsymbol{n}}u(\boldsymbol{x}) = 0$ for $\boldsymbol{x}\in \partial \Omega $. Additionally, let $\boldsymbol{C}=\operatorname{diag}(\boldsymbol{c})$ be the diagonal matrix with the mask vector $\boldsymbol{c}\in \{0,1\}^{N}$ discretizing *c*, and let ***I*** be the $N \times N$ identity matrix. Then the discrete version of (13) can be formulated as the linear system of equations,

14 $$ \left (\boldsymbol{C}+(\boldsymbol{I}-\boldsymbol{C})\boldsymbol{L}\right )\boldsymbol{u} = \boldsymbol{C}\boldsymbol{f}, $$

and the reconstruction can be written explicitly as

15 $$ \boldsymbol{u} = \boldsymbol{r}(\boldsymbol{c}, \boldsymbol{f}) = \left (\boldsymbol{C}+(\boldsymbol{I}-\boldsymbol{C})\boldsymbol{L} \right )^{-1}\boldsymbol{C}\boldsymbol{f}. $$

The inverse of the *inpainting matrix* $\boldsymbol{M}_{\boldsymbol{c}} :=\boldsymbol{C}+(\boldsymbol{I}-\boldsymbol{C})\boldsymbol{L}$ exists as long as $\boldsymbol{C}\ne \boldsymbol{0}$ [33]. To deal with the case $\boldsymbol{C}=\boldsymbol{0}$, we define $\boldsymbol{r}(\boldsymbol{0},\boldsymbol{f}) :=\frac{1}{N} \boldsymbol{1}^{\mathsf{T}} \boldsymbol{f}$, i.e., we take the average. If we want to approximate the image ***f*** instead of interpolating it over ***C***, we can replace ***Cf*** with ***Cg***, where

16 $$ \boldsymbol{g} \in \operatorname*{\textrm{argmin}}_{\boldsymbol{h}\,:\,\boldsymbol{h}|_{\bar{\boldsymbol{c}}} = \boldsymbol{0}} \| \boldsymbol{r}(\boldsymbol{c},\boldsymbol{h})-\boldsymbol{f}\|^{2}_{2}. $$

Here $\boldsymbol{h}|_{\boldsymbol{c}}$ is the restriction of ***h*** to ***c*** and $\boldsymbol{h}|_{\bar{\boldsymbol{c}}}$ is the restriction of ***h*** to the complement $\bar{\boldsymbol{c}} = \boldsymbol{1}-\boldsymbol{c}$. The optimization is thus only over $\boldsymbol{h}|_{\boldsymbol{c}}$ since the remainder of the values are irrelevant for the inpainting result, so we set them to zero. The least squares problem is known as the *tonal optimization* problem and we discuss its implications for the current work in Sect. 3.1.1. Additionally, we observe that the reconstruction is linear in ***g***. This motivates us to write it as a linear combination of basis vectors with weights given by $\boldsymbol{g}|_{\boldsymbol{c}}$. Let $\boldsymbol{B}_{\boldsymbol{c}} :=(\boldsymbol{M}_{\boldsymbol{c}}^{-1})|_{\boldsymbol{I}\times \boldsymbol{C}}$ be the restriction of $\boldsymbol{M}^{-1}_{\boldsymbol{c}}$ to the columns corresponding to nonzeros in ***c***, and we set $m=\|\boldsymbol{c}\|_{0}$ to be the number of nonzeros in ***c***. By denoting the columns as $\left \{\boldsymbol{b}_{\boldsymbol{c}}^{k}\right \}_{k=1}^{m}$, i.e., $\boldsymbol{B}_{\boldsymbol{c}} = \left [\boldsymbol{b}_{\boldsymbol{c}}^{1} \, \ldots \, \boldsymbol{b}_{ \boldsymbol{c}}^{m} \right ]$, we can write the reconstruction as

17 $$ \boldsymbol{u} = \boldsymbol{r}(\boldsymbol{c}, \boldsymbol{g}) = \boldsymbol{M}^{-1}_{\boldsymbol{c}}\boldsymbol{C}\boldsymbol{g} = \boldsymbol{B}_{\boldsymbol{c}}\,\boldsymbol{g}|_{\boldsymbol{c}} = \sum _{k=1}^{m} (\boldsymbol{g}|_{\boldsymbol{c}})_{k} \,\boldsymbol{b}^{k}_{\boldsymbol{c}}. $$

We see that the columns of $\boldsymbol{B}_{\boldsymbol{c}}$ are the basis vectors induced from ***r*** and ***c***. They are also termed *inpainting echoes* [39, 90]. We note that inpainting with $\boldsymbol{g}|_{\boldsymbol{c}} = \boldsymbol{f}|_{\boldsymbol{c}}$ constructs the interpolant over ***c*** in the space $\operatorname{span}(\boldsymbol{B}_{\boldsymbol{c}}) \subseteq \mathbb{R}^{N}$. Since the tonal optimization solution can be written as $\boldsymbol{g}|_{\boldsymbol{c}} = (\boldsymbol{B}_{\boldsymbol{c}})^{+}\boldsymbol{f}$, where $(\boldsymbol{B}_{\boldsymbol{c}})^{+}$ is the Moore–Penrose pseudoinverse, we note that $\boldsymbol{r}(\boldsymbol{c},\boldsymbol{g}) = \boldsymbol{B}_{\boldsymbol{c}}(\boldsymbol{B}_{\boldsymbol{c}})^{+}\boldsymbol{f}$ is the orthogonal projection of ***f*** on the subspace $\operatorname{span}(\boldsymbol{B}_{\boldsymbol{c}}) \subseteq \mathbb{R}^{N}$, i.e., the best approximant of ***f*** in this space.

## Our denoising by inpainting framework

We now present the basic idea and the framework for denoising by inpainting proposed in our conference paper [10]. Since the framework inherently links inpainting and denoising, it is well suited to study connections between the two tasks. As previously mentioned, we use diffusion-based inpainting – specifically homogeneous diffusion inpainting – for image denoising, by only keeping a sparse subset of the noisy input data and by reconstructing the rest. Inpainting on noisy images differs from the classical setting and poses additional challenges. During the inpainting process, gray values at mask locations are not altered. As they might contain errors from the noise, these mask pixels are less trustworthy than inpainted pixels, which combine information from their surrounding mask pixels. While we want to exploit the filling-in effect in unknown areas, this observation implies that a single inpainted image cannot give satisfactory denoising results. Therefore, we compute multiple inpaintings with different masks and obtain the final result by averaging them. This ensures that none of the pixels remain unchanged (unless a pixel is contained in all masks). In the current work, we further mitigate the issue of noisy mask pixels by employing tonal optimization (see Sect. 3.1.1). If we denote the *n* different masks by $\{\boldsymbol{c}^{\ell}\}_{\ell =1}^{n}$, we can generate the inpaintings $\{\boldsymbol{v}^{\ell}\}_{\ell =1}^{n}$ via

18 $$ \boldsymbol{v}^{\ell }= \boldsymbol{r}(\boldsymbol{c}^{\ell}, \boldsymbol{f}) = \left (\boldsymbol{C}^{\ell}+ \left (\boldsymbol{I}-\boldsymbol{C}^{\ell}\right )\boldsymbol{L}\right )^{-1} \boldsymbol{C}^{\ell} \boldsymbol{f}. $$

We obtain the final denoising result $\langle \boldsymbol{u}\rangle _{n}$ by averaging,

19 $$ \langle \boldsymbol{u} \rangle _{n}= \frac{1}{n} \sum _{\ell =1}^{n} \boldsymbol{v}^{ \ell }= \frac{1}{n}\sum _{\ell =1}^{n}\boldsymbol{r}(\boldsymbol{c}^{\ell},\boldsymbol{f}). $$

As we fix the inpainting operator (for a discussion of denoising by biharmonic inpainting see Sect. 6.4), the only freedom in the framework lies in the selection of the different masks. This is in contrast to the common strategy in denoising, where all available data is used and the operator is optimized instead. To study the effects of different data selection strategies, we will borrow several ideas from mask optimization for image compression. To obtain multiple different masks as our framework requires, we rely on some degree of randomness in the mask generation processes (see Sect. 5). Since we make use of stochastic strategies, we formalize and study DbI from a probabilistic point of view in the following subsection.

### Probabilistic theory

As seen in (19), the denoised image is the result of averaging *n* inpaintings from *n* different masks, that are generated by some mask optimization process. In the following, we interpret this from a probabilistic point of view. This allows us to formalize the DbI framework from our conference paper [10] and provides us with tools to study and boost the convergence of our methods in Sects. 3.1.4 and 3.1.5, respectively. We take the masks $\{\boldsymbol{c}^{\ell}\}_{\ell =1}^{n}$ to be independent and identically distributed samples from a predetermined distribution conditioned on ***f***, with a conditional probability mass function (PMF) $p(\boldsymbol{c}|\boldsymbol{f})$. Then the estimator ***u*** converges to the following conditional expectation for $n\to \infty $:

20 $$ \mathbb{E}[\langle \boldsymbol{u}\rangle _{n}|\boldsymbol{f}] = \mathbb{E}\left [ \frac{1}{n} \sum _{\ell =1}^{n}\boldsymbol{r}(\boldsymbol{c}^{\ell},\boldsymbol{f})\biggr| \boldsymbol{f}\right ] = \frac{1}{n}\sum _{\ell =1}^{n}\mathbb{E}[\boldsymbol{r}( \boldsymbol{c},\boldsymbol{f})|\boldsymbol{f}] = \sum _{\boldsymbol{c}\in \{0,1\}^{N}}\boldsymbol{r}(\boldsymbol{c}, \boldsymbol{f})\,p(\boldsymbol{c}|\boldsymbol{f}). $$

The second equality holds because the masks were assumed to be identically distributed, and thus $\mathbb{E}[\boldsymbol{r}(\boldsymbol{c}^{\ell},\boldsymbol{f})|\boldsymbol{f}] = \mathbb{E}[\boldsymbol{r}( \boldsymbol{c},\boldsymbol{f})|\boldsymbol{f}]$ for any ***c*** sampled with the same PMF *p*. The third equality follows from the definition of the conditional mathematical expectation. We note that from this probabilistic point of view, spatial adaptivity is provided through the design of the PMF *p*. The following proposition shows that the DbI result constitutes a minimum mean squared error (MMSE) estimate. This emphasizes its optimality under certain assumptions.

#### Proposition 1

(DbI as an MMSE Estimate)

*The expectation* (20) *of the DbI averaging* (19) *can be interpreted as an MMSE estimate under prior assumptions on the image and noise distributions*, *i*.*e*., *it solves the minimization problem*

21 $$ \min _{\boldsymbol{u}\in \mathbb{R}^{N}} \mathbb{E}[\|\boldsymbol{u}-\boldsymbol{w}\|^{2}_{2}| \boldsymbol{f}] = \min _{\boldsymbol{u}\in \mathbb{R}^{N}} \mathbb{E}[\|\boldsymbol{u}-\boldsymbol{r}( \boldsymbol{c},\boldsymbol{f})\|^{2}_{2}|\boldsymbol{f}]. $$

#### Proof

We can rewrite the minimization problem (21) as

22 $$ \min _{\boldsymbol{u}\in \mathbb{R}^{N}} \mathbb{E}[\|\boldsymbol{u}-\boldsymbol{r}(\boldsymbol{c}, \boldsymbol{f})\|^{2}_{2}|\boldsymbol{f}] = \min _{\boldsymbol{u}\in \mathbb{R}^{N}} \sum _{ \boldsymbol{c}\in \{0,1\}^{N}} \|\boldsymbol{u}-\boldsymbol{r}(\boldsymbol{c},\boldsymbol{f})\|^{2}_{2}\,p( \boldsymbol{c}|\boldsymbol{f}). $$

Taking the derivative with respect to ***u*** and setting it to zero results in the MMSE estimate

23 $$ \boldsymbol{u}^{\text{MMSE}} = \mathbb{E}[\boldsymbol{r}(\boldsymbol{c},\boldsymbol{f})|\boldsymbol{f}] = \sum _{\boldsymbol{c}\in \{0,1\}^{N}}\boldsymbol{r}(\boldsymbol{c}, \boldsymbol{f})\,p(\boldsymbol{c}|\boldsymbol{f}). $$

By (20) this is the same as the expectation $\mathbb{E}[\langle \boldsymbol{u}\rangle _{n}]$ of the DbI estimator $\langle \boldsymbol{u}\rangle _{n}$. □

The estimate $\boldsymbol{u}^{\text{MMSE}}$ is close to $\boldsymbol{f}_{r}$ (and $\langle \boldsymbol{u}\rangle _{n}$ is close to $\boldsymbol{f}_{r}$), whenever $\boldsymbol{v}=\boldsymbol{r}(\boldsymbol{c},\boldsymbol{f})$ with $\boldsymbol{c}\sim p(\boldsymbol{c}|\boldsymbol{f})$ provides a good model for the distribution from which $\boldsymbol{f}_{r}$ is assumed to originate. This formalization of DbI as an estimator for the MMSE estimate therefore provides an additional justification for the DbI framework as an image denoising approach.

#### MMSE and tonal optimization

The classical DbI formulation (19) from [10] employs an *interpolating* inpainting. It is natural to extend the framework to the best *approximating* inpainting, computing the denoised image $\langle \boldsymbol{u}\rangle _{n}$ as

24 $$ \langle \boldsymbol{u}\rangle _{n} = \frac{1}{n}\sum _{\ell =1}^{n}\boldsymbol{r}( \boldsymbol{c}^{\ell},\boldsymbol{g^{\ell}}), $$

where the masks $\{\boldsymbol{c}^{\ell}\}_{\ell =1}^{n}$ are selected as before, while $\{\boldsymbol{g}^{\ell}\}_{\ell =1}^{n}$ are the solutions to the corresponding tonal optimization problems (16). Next we show that after relaxing assumptions on the gray values compared to Proposition 1, the MMSE estimate actually corresponds to DbI with an approximating inpainting instead of an interpolating one.

##### Proposition 2

(DbI with Approximating Inpainting as an MMSE Estimate)

*The DbI result based on a best approximating inpainting* (24) *can also be interpreted as an MMSE estimate*, *assuming that the gray values* ***h*** *are now also a random variable conditioned on ****f***.

##### Proof

Firstly, we note that the minimization problem for the MMSE now differs, as the expectation has to be taken over the gray values ***h*** as well:

25 $$ \begin{aligned} \min _{\boldsymbol{u}\in \mathbb{R}^{N}} \mathbb{E}[\|\boldsymbol{u}- \boldsymbol{w}\|^{2}_{2}|\boldsymbol{f}] =&\min _{\boldsymbol{u}\in \mathbb{R}^{N}} \mathbb{E}[\|\boldsymbol{u}-\boldsymbol{r}(\boldsymbol{c},\boldsymbol{h})\|^{2}_{2}|\boldsymbol{f}] \\ = &\min _{\boldsymbol{u}\in \mathbb{R}^{N}} \sum _{\boldsymbol{c}\in \{0,1\}^{N}} \mathbb{E}[\|\boldsymbol{u}-\boldsymbol{r}(\boldsymbol{c},\boldsymbol{h})\|^{2}_{2}|\boldsymbol{f},\boldsymbol{c}] \, p( \boldsymbol{c}|\boldsymbol{f}) \\ = &\min _{\boldsymbol{u}\in \mathbb{R}^{N}} \sum _{\boldsymbol{c}\in \{0,1\}^{N}} \left (\int _{\boldsymbol{h}\in \mathbb{R}^{N}} \|\boldsymbol{u}-\boldsymbol{r}(\boldsymbol{c},\boldsymbol{h}) \|^{2}_{2} \,p(\boldsymbol{h}|\boldsymbol{f},\boldsymbol{c})\,d\boldsymbol{h}\right ) p(\boldsymbol{c}|\boldsymbol{f}). \end{aligned} $$

As before, differentiation with respect to ***u*** yields the MMSE estimate

26 $$ \boldsymbol{u}^{\text{MMSE}} = \mathbb{E}[\boldsymbol{r}(\boldsymbol{c},\boldsymbol{h})|\boldsymbol{f}] = \sum _{\boldsymbol{c}\in \{0,1\}^{N}} \mathbb{E}[\boldsymbol{r}(\boldsymbol{c},\boldsymbol{h})|\boldsymbol{f}, \boldsymbol{c}] \, p(\boldsymbol{c}|\boldsymbol{f}), $$

which is similar to (23), but now contains the expectation

27 $$ \mathbb{E}[\boldsymbol{r}(\boldsymbol{c},\boldsymbol{h})|\boldsymbol{f},\boldsymbol{c}] = \int _{\boldsymbol{h}\in \mathbb{R}^{N}} \boldsymbol{r}(\boldsymbol{c},\boldsymbol{h})\,p(\boldsymbol{h}|\boldsymbol{f},\boldsymbol{c})\,d \boldsymbol{h}. $$

To compute $\mathbb{E}[\boldsymbol{r}(\boldsymbol{c},\boldsymbol{h})|\boldsymbol{f},\boldsymbol{c}]$, we need to know the a posteriori density $p(\boldsymbol{h}|\boldsymbol{f},\boldsymbol{c})$. If we assume that the noise is normally distributed $\boldsymbol{n} = (\boldsymbol{r}(\boldsymbol{c},\boldsymbol{h})-\boldsymbol{f}) \sim \mathcal{N}(\boldsymbol{0}, \sigma ^{2}_{\boldsymbol{n}}\boldsymbol{I})$, and that the gray values restricted to the mask $\boldsymbol{h}|_{\boldsymbol{c}}$ are normally distributed $\boldsymbol{h}|_{\boldsymbol{c}}\sim \mathcal{N}(\boldsymbol{0}, \sigma ^{2}_{\boldsymbol{h}|_{ \boldsymbol{c}}}\boldsymbol{I})$, then the expectation can be calculated [82] as

28 $$ \mathbb{E}[\boldsymbol{r}(\boldsymbol{c},\boldsymbol{h})|\boldsymbol{f},\boldsymbol{c}] = \boldsymbol{B}_{\boldsymbol{c}}\, \mathbb{E}[\boldsymbol{h}|_{\boldsymbol{c}}|\boldsymbol{f},\boldsymbol{c}] = \boldsymbol{B}_{\boldsymbol{c}} \left ( \frac{\sigma ^{2}_{\boldsymbol{n}}}{\sigma _{\boldsymbol{h}|_{\boldsymbol{c}}}^{2}}\boldsymbol{I} + \boldsymbol{B}_{\boldsymbol{c}}^{\mathsf{T}}\boldsymbol{B}_{\boldsymbol{c}}\right )^{-1}\boldsymbol{B}_{ \boldsymbol{c}}^{\mathsf{T}} \boldsymbol{f}. $$

Since we do not know $\sigma _{\boldsymbol{h}|_{\boldsymbol{c}}}$ and because the assumption of the normality of the gray values may not be a very plausible one, we can dispense away with it by taking $\sigma _{\boldsymbol{h}|_{\boldsymbol{c}}}\to \infty $, which results in a tonally optimized inpainting:

29 $$ \lim _{\sigma _{\boldsymbol{h}|_{\boldsymbol{c}}}\to \infty} \mathbb{E}[\boldsymbol{r}(\boldsymbol{c}, \boldsymbol{h})|\boldsymbol{f},\boldsymbol{c}] = \boldsymbol{B}_{\boldsymbol{c}}\lim _{\sigma _{\boldsymbol{h}|_{ \boldsymbol{c}}}\to \infty} \left ( \frac{\sigma ^{2}_{\boldsymbol{n}}}{\sigma _{\boldsymbol{h}|_{\boldsymbol{c}}}^{2}}\boldsymbol{I} + \boldsymbol{B}_{\boldsymbol{c}}^{\mathsf{T}}\boldsymbol{B}_{\boldsymbol{c}}\right )^{-1}\boldsymbol{B}_{ \boldsymbol{c}}^{\mathsf{T}} \boldsymbol{f} = \boldsymbol{B}_{\boldsymbol{c}} (\boldsymbol{B}_{\boldsymbol{c}})^{+} \boldsymbol{f}. $$

Using $\boldsymbol{B}_{\boldsymbol{c}}(\boldsymbol{B}_{\boldsymbol{c}})^{+}\boldsymbol{f} = \boldsymbol{r}(\boldsymbol{c}, (\boldsymbol{B}_{ \boldsymbol{c}})^{+}\boldsymbol{f})$, the new MMSE estimate differs with (23) only in that we have approximation instead of interpolation:

30 $$ \boldsymbol{u}^{\text{MMSE}} = \sum _{\boldsymbol{c}\in \{0,1\}^{N}} \mathbb{E}[\boldsymbol{r}( \boldsymbol{c},\boldsymbol{h})|\boldsymbol{f},\boldsymbol{c}] \, p(\boldsymbol{c}|\boldsymbol{f}) = \sum _{\boldsymbol{c}\in \{0,1\}^{N}} \boldsymbol{r}(\boldsymbol{c},(\boldsymbol{B}_{\boldsymbol{c}})^{+}\boldsymbol{f}) \, p(\boldsymbol{c}| \boldsymbol{f}). $$

This corresponds exactly to the expectation of the approximating DbI formulation. □

We note that the above analysis did not require ***r*** to be linear in ***f*** except for the approximation of ***f***. Given a fixed ***c***, a natural extension to nonlinear operators could use nonlinear least-squares to compute something similar to $\boldsymbol{B}_{\boldsymbol{c}}^{+}\boldsymbol{f}$. By using the approximating formulation, we project the image onto the various subspaces induced by the inpainting operator ***r*** and the mask ***c***. We will show in Sect. 6.3.2 that in practice, tonal optimization is able to improve quality and to reduce the variance of MMSE denoising, since it mitigates the error from the interpolation of noisy mask pixels and provides representations that are closer to ***f*** in terms of MSE.

#### Interpreting tonal optimization as MAP estimate

Not directly related to the classical averaging formulation of DbI, but nevertheless interesting and a valuable extension, is the fact that spatial and tonal optimization for a single inpainting can also be framed as a maximum a posteriori (MAP) estimate. In MAP estimation, instead of minimizing the MSE, we want to find an inpainting ***w*** that maximizes the posterior:

31 $$ \operatorname*{\textrm{argmax}}_{\boldsymbol{w}} p(\boldsymbol{w}|\boldsymbol{f}) = \operatorname*{\textrm{argmax}}_{\boldsymbol{c},\boldsymbol{h}} p( \boldsymbol{h},\boldsymbol{c}|\boldsymbol{f}) = \operatorname*{\textrm{argmax}}_{\boldsymbol{c},\boldsymbol{h}} p(\boldsymbol{f}|\boldsymbol{h}, \boldsymbol{c}) p(\boldsymbol{h}|\boldsymbol{c}) p(\boldsymbol{c}). $$

We have assumed that $\boldsymbol{w}=\boldsymbol{r}(\boldsymbol{c},\boldsymbol{h})$ is an injection, so we have $p(\boldsymbol{w}|\boldsymbol{f}) = p(\boldsymbol{r}(\boldsymbol{c},\boldsymbol{h})|\boldsymbol{f}) = p(\boldsymbol{h},\boldsymbol{c}| \boldsymbol{f})$. In the noninjective case, one gets a set

32 $$ p(\boldsymbol{w}|\boldsymbol{f}) = p(\boldsymbol{r}^{-1}(\boldsymbol{w})|\boldsymbol{f}) = p(\{\boldsymbol{h},\boldsymbol{c} \,:\,\boldsymbol{w}=\boldsymbol{r}(\boldsymbol{c},\boldsymbol{h})\}|\boldsymbol{f}), $$

which does not change the derivation meaningfully, except for introducing additional technical details. Thus, for the sake of clarity, we proceed with the injective case, but a similar argument holds in the general setting. The maximization problem (31) can be split into two optimization problems:

33 $$ \max _{\boldsymbol{c},\boldsymbol{h}} p(\boldsymbol{f}|\boldsymbol{h},\boldsymbol{c}) p(\boldsymbol{h}|\boldsymbol{c}) p( \boldsymbol{c}) = \max _{\boldsymbol{c}}\left (\max _{\boldsymbol{h}} p(\boldsymbol{f}|\boldsymbol{h},\boldsymbol{c}) p( \boldsymbol{h}|\boldsymbol{c})\right )p(\boldsymbol{c}). $$

The inner one optimizes over the gray values ***h*** given a mask ***c***, and the outer one optimizes over the masks ***c***. If we again assume that $\boldsymbol{f} = \boldsymbol{r}(\boldsymbol{c},\boldsymbol{h}) + \boldsymbol{n}$, where $\boldsymbol{n}\sim \mathcal{N}(\boldsymbol{0}, \sigma ^{2}_{\boldsymbol{n}}\boldsymbol{I})$, then the density $p(\boldsymbol{f}|\boldsymbol{h}, \boldsymbol{c})$ is given by a Gaussian

34 $$ p(\boldsymbol{f}|\boldsymbol{h}, \boldsymbol{c}) = \frac{1}{(2\pi \sigma _{\boldsymbol{n}}^{2})^{N/2}} \exp \left (- \frac{\|\boldsymbol{r}(\boldsymbol{c},\boldsymbol{h})-\boldsymbol{f}\|^{2}_{2}}{\sigma _{\boldsymbol{n}}^{2}}\right ). $$

Assuming also that the gray values are normally distributed, i.e., $\boldsymbol{h}|_{\boldsymbol{c}}\sim \mathcal{N}(\boldsymbol{0},\sigma ^{2}_{\boldsymbol{h}|_{\boldsymbol{c}}} \boldsymbol{I})$, then the minimization problem with respect to ***h*** is what we call *the regularized tonal optimization problem*:

35 $$ \operatorname*{\textrm{argmax}}_{\boldsymbol{h}|_{\bar{\boldsymbol{c}}}=\boldsymbol{0}} \exp \left (- \frac{\|\boldsymbol{r}(\boldsymbol{c},\boldsymbol{h})-\boldsymbol{f}\|^{2}_{2}}{\sigma _{\boldsymbol{n}}^{2}} - \frac{\|\boldsymbol{h}|_{\boldsymbol{c}}\|^{2}_{2}}{\sigma ^{2}_{\boldsymbol{h}|_{\boldsymbol{c}}}} \right ) = \operatorname*{\textrm{argmin}}_{\boldsymbol{h}|_{\bar{\boldsymbol{c}}}=\boldsymbol{0}} \|\boldsymbol{B}_{\boldsymbol{c}} \boldsymbol{h}|_{\boldsymbol{c}} - \boldsymbol{f}\|^{2}_{2} + \frac{\sigma _{\boldsymbol{n}}^{2}}{\sigma ^{2}_{\boldsymbol{h}|_{\boldsymbol{c}}}} \|\boldsymbol{h}_{ \boldsymbol{c}}\|^{2}_{2}, $$

where the solution is the same as in (28), namely

36 $$ \boldsymbol{h}|^{*}_{\boldsymbol{c}} = \left ( \frac{\sigma ^{2}_{\boldsymbol{n}}}{\sigma _{\boldsymbol{h}|_{\boldsymbol{c}}}^{2}}\boldsymbol{I} + \boldsymbol{B}_{\boldsymbol{c}}^{\mathsf{T}}\boldsymbol{B}_{\boldsymbol{c}}\right )^{-1} \boldsymbol{B}_{ \boldsymbol{c}}^{\mathsf{T}} \boldsymbol{f}. $$

Note that this can already be used for denoising with just a single inpainting with a mask ***c***, provided that we know the ratio of the variances of the noise and the gray values. The above expression suggests that we can then just apply a regularized tonal optimization to get the best MAP estimate. As before, we may take $\sigma _{\boldsymbol{h}|_{\boldsymbol{c}}}\to \infty $ to get classical tonal optimization if desired. Of course, we also need to optimize with respect to the masks according to $p(\boldsymbol{c})$. In fact, if we take $p(\boldsymbol{c}) = 0$ for $\|\boldsymbol{c}\|_{0}\ne m$, and $p(\boldsymbol{c})$ being equal for all $\|\boldsymbol{c}\|_{0}=m$, then we get the spatial optimization problem with tonally optimized values

37 $$ \min _{\|\boldsymbol{c}\|_{0} = m} \|\boldsymbol{r}(\boldsymbol{c},\boldsymbol{h}|^{*}_{c}(\boldsymbol{f})) - \boldsymbol{f}\|^{2}_{2}. $$

If we take the interpolating case, we get the classical spatial optimization problem [39]

38 $$ \min _{\|\boldsymbol{c}\|_{0}=m} \|\boldsymbol{r}(\boldsymbol{c},\boldsymbol{f}) - \boldsymbol{f}\|^{2}_{2}. $$

The above further motivates using spatial optimization for denoising in both the interpolation and approximation cases; see Sect. 5.

#### Bayesian interpretation

In this subsection, we discuss how the above approaches fit in a general Bayesian perspective, which allows for meaningful interpretations of the occurring probabilities. This is valuable as MMSE and MAP estimates rely on a posterior $p(\boldsymbol{w}|\boldsymbol{f})$. Using Bayes’ rule, this posterior can be rewritten as

39 $$ p(\boldsymbol{w}|\boldsymbol{f}) = \frac{p(\boldsymbol{f}|\boldsymbol{w}) p(\boldsymbol{w})}{p(\boldsymbol{f})} = \frac{p(\boldsymbol{f}|\boldsymbol{w}) p(\boldsymbol{w})}{\int _{\mathbb{R}^{N}}p(\boldsymbol{f}|\boldsymbol{w}) p(\boldsymbol{w})\,d\boldsymbol{w}}, $$

where $p(\boldsymbol{w})$ is the probability density function (PDF) for the distribution of images ***w*** from which we assume $\boldsymbol{f}_{r}$ to originate. The likelihood $p(\boldsymbol{f}|\boldsymbol{w})$ is the noise PDF, which in our case is a Gaussian. The term $p(\boldsymbol{f})$ is just a normalization constant that is irrelevant in practice, since it is not a function of ***w***. This shows that the task of finding a proper posterior distribution corresponds to introducing an appropriate prior $p(\boldsymbol{w})$ under a given noise distribution $p(\boldsymbol{f}|\boldsymbol{w})$. This is known to be crucial for good denoising performance of Bayesian methods, and links our DbI framework to such approaches.

##### Incorporating the inpainting operator

To introduce an inpainting operator ***r*** into the above model, we make the assumption that any ***w*** is synthesized as $\boldsymbol{w} = \boldsymbol{r}(\boldsymbol{c},\boldsymbol{h})$ for some mask ***c*** and some gray values $\boldsymbol{h}|_{\boldsymbol{c}}$. Since now the model depends on the masks we can rewrite the PDF as

40 $$ p(\boldsymbol{w}|\boldsymbol{f}) = \sum _{\boldsymbol{c}\in \{0,1\}^{N}} p(\boldsymbol{w}|\boldsymbol{f}, \boldsymbol{c}) p(\boldsymbol{c}|\boldsymbol{f}), $$

which is where the conditional mask PMF $p(\boldsymbol{c}|\boldsymbol{f})$ comes into play – this is the other key ingredient for DbI along with the inpainting operator. We will see that this PMF allows us to introduce spatial adaptivity (Sect. 5.2, Fig. 9) for operators that are otherwise not spatially adaptive. Finally, we can also rewrite $p(\boldsymbol{w}|\boldsymbol{f},\boldsymbol{c})$ using Bayes’ rule in order to relate the above formulation to (39):

41 $$ p(\boldsymbol{w}|\boldsymbol{f},\boldsymbol{c}) = \frac{p(\boldsymbol{f}|\boldsymbol{w},\boldsymbol{c})p(\boldsymbol{w}|\boldsymbol{c})}{p(\boldsymbol{f}|\boldsymbol{c})} = \frac{p(\boldsymbol{f}|\boldsymbol{w},\boldsymbol{c})p(\boldsymbol{w}|\boldsymbol{c})}{\int _{\mathbb{R}^{N}}p(\boldsymbol{f}|\boldsymbol{w},\boldsymbol{c})p(\boldsymbol{w}|\boldsymbol{c})\,d\boldsymbol{w}}. $$

This provides a similar interpretation, but now we have knowledge about the mask. As before $p(\boldsymbol{f}|\boldsymbol{w},\boldsymbol{c})$ models the noise, but now $p(\boldsymbol{w}|\boldsymbol{c})$ models the distribution of the gray values defining ***w*** given ***c***, i.e., the distribution of $\boldsymbol{h}|_{\boldsymbol{c}}$. As before, the denominator is a normalization constant that is not practically relevant.

##### The mask posterior

Bayes’ rule allows us to explore further theoretical considerations about the involved mask probabilities. We can study the mask posterior $p(\boldsymbol{c}|\boldsymbol{f})$ in more detail, using

42 $$ p(\boldsymbol{c}|\boldsymbol{f}) = \frac{p(\boldsymbol{f}|\boldsymbol{c})p(\boldsymbol{c})}{p(\boldsymbol{f})}. $$

Now $p(\boldsymbol{c})$ models the probability of the mask ***c*** being generated (irrespective of ***f***) and $p(\boldsymbol{f}|\boldsymbol{c})$ models some measure of the noise and image content in relation to the mask. In practice, ideally the density $\boldsymbol{1}^{\mathsf{T}}\mathbb{E}[\boldsymbol{c}]/N$ should be chosen to be inversely proportional to the standard deviation of the noise. Similarly if we know that the noise distribution is space-variant, or if we suspect that features (e.g., edges) are present, we can choose the local density of ***c*** to account for that: higher for more prominent edges, lower for higher noise variance. The weight of these choices are modeled by $p(\boldsymbol{f}|\boldsymbol{c})$. Selecting $p(\boldsymbol{c})$ is less trivial, as it needs to match the mask distribution of natural images, i.e., the distribution of natural images from the perspective of the masks used in the inpainting operator. It is simpler to choose it based on the density, i.e., $p(\boldsymbol{c}) = p(\|\boldsymbol{c}\|_{0}/N)$, which makes it blind to spatial variations, or to just choose it as a constant, if we have no data on it. Note that these considerations are meant to provide a different view on the mask posterior and an alternative strategy on how to construct it. The adaptive mask selection methods that we consider in this work directly induce a mask posterior $p(\boldsymbol{c}|\boldsymbol{f})$ and do not model $p(\boldsymbol{f}|\boldsymbol{c})$ or $p(\boldsymbol{c})$. They are based on strategies from the noise-free case in image inpainting and we adapt and extend them to the noisy case. For all the approaches that we consider, we state their induced PMFs $p(\boldsymbol{c}|\boldsymbol{f})$ (see Eq. (64), Proposition 5, Appendix B).

##### On the importance of the inpainting operator

A crucial question is whether an inpainting operator ***r*** is suitable for modeling natural images in a sparse and robust manner, such that noise can be attenuated by averaging multiple nearby representations of a noisy image from a lower-dimensional image manifold. For ***r*** being homogeneous diffusion inpainting, we know that it has been used successfully for image compression of natural images with low to medium frequencies [33]. Moreover, we present new results in Sect. 4 that relate the MMSE estimate to homogeneous diffusion denoising. The large body of literature on sparse image approximation and compression should provide a reasonable selection of good inpainting operators ***r***. In the current work we also consider biharmonic inpainting (see Sect. 6.4).

##### Interplay between the mask PMF and homogeneous diffusion

The basis vectors $\boldsymbol{B}_{\boldsymbol{c}}$ for homogeneous diffusion are generally low-frequent and smooth, with the local frequency depending on the local density of the mask points. For a constant PMF $p(\boldsymbol{c}|\boldsymbol{f})$, i.e., a homogeneous mask density, we get a process similar to isotropic homogeneous diffusion, and it is in fact approximately equivalent to it, as we demonstrate later in Sects. 4.2 and 4.3. As such it also shares its drawbacks, i.e., smoothing equally over image structures and noise. More sophisticated denoising methods such as space-variant diffusion allow for steering the smoothing away from image structures by relying on a guidance image, e.g., the gradient magnitude $|\boldsymbol{\nabla}u|$. Similarly, we may use the PMF $p(\boldsymbol{c}|\boldsymbol{f})$ to guide the denoising. One instance of a PMF that we consider is inspired by a result for mask selection in inpainting. Belhachmi et al. [20] have argued that the local density of an optimal inpainting mask ***c*** should be proportional to the pixelwise magnitude of the Laplacian $|\boldsymbol{L}\boldsymbol{f}|$. In our setting this translates to constructing a PMF *p* such that $\mathbb{E}[\boldsymbol{c}|\boldsymbol{f}]\sim |\boldsymbol{L}\boldsymbol{f}|$; see Sect. 5.2.

#### Convergence

A question which arises is how well the estimator $\langle \boldsymbol{u}\rangle _{n}$ approximates the MMSE estimate $\boldsymbol{u}^{\text{MMSE}} = \mathbb{E}[\langle \boldsymbol{u}\rangle _{n}|\boldsymbol{f}]$ as a function of the number of samples *n*. We consider this scaling behavior in the next proposition.

##### Proposition 3

(Convergence of the DbI Estimator)

*The root mean square error* (*RMSE*) $\sqrt{\operatorname{MSE}(\langle \boldsymbol{u}\rangle _{n}, \mathbb{E}[ \langle \boldsymbol{u}\rangle _{n}|\boldsymbol{f}])}$ *between the estimator* $\langle \boldsymbol{u}\rangle _{n}$ *and its expectation* $\mathbb{E}[\langle \boldsymbol{u}\rangle _{n}|\boldsymbol{f}]$ *scales as* $O(n^{-1/2})$, *where* *n* *is the number of sampled masks*.

##### Proof

We first recall that we can decompose the MSE between some estimator $\hat{\boldsymbol{\theta}}$ and some fixed parameter ***θ*** into a variance and a bias part:

43 $$ \begin{aligned} \text{MSE}(\hat{\boldsymbol{\theta}}, \boldsymbol{\theta}) &= \mathbb{E} \bigl[\|\hat{\boldsymbol{\theta}} - \boldsymbol{\theta}\|^{2}_{2}\bigr] \\ &= \mathbb{E}\bigl[\|\hat{\boldsymbol{\theta}} - \mathbb{E}[\hat{\boldsymbol{\theta}}] \|^{2}_{2}\bigr] + \|\mathbb{E}[\hat{\boldsymbol{\theta}}] - \boldsymbol{\theta}\|^{2}_{2} \\ &= \mathbb{V}[\hat{\boldsymbol{\theta}}] + \operatorname{Bias}( \hat{\boldsymbol{\theta}}, \boldsymbol{\theta})^{2}. \end{aligned} $$

If we consider the MSE between the estimator $\langle \boldsymbol{u}\rangle _{n}$ and its expectation $\mathbb{E}[\langle \boldsymbol{u}\rangle _{n}|\boldsymbol{f}]$, the bias vanishes and we have $\text{MSE}(\langle \boldsymbol{u}\rangle _{n}, \mathbb{E}[\langle \boldsymbol{u} \rangle _{n}|\boldsymbol{f}]) = \mathbb{V}[\langle \boldsymbol{u}\rangle _{n}|\boldsymbol{f}]$. The variance $\mathbb{V}[\langle \boldsymbol{u}\rangle _{n}|\boldsymbol{f}]$ is given by

44 $$ \mathbb{V}[\langle \boldsymbol{u}\rangle _{n}|\boldsymbol{f}] = \mathbb{V}\left [ \frac{1}{n}\sum _{\ell =1}^{n} \boldsymbol{r}(\boldsymbol{c}^{\ell},\boldsymbol{f})\biggr| \boldsymbol{f}\right ] = \frac{1}{n^{2}}\sum _{\ell =1}^{n} \mathbb{V}\left [ \boldsymbol{r}(\boldsymbol{c},\boldsymbol{f})|\boldsymbol{f}\right ] = \frac{1}{n}\mathbb{V}[\boldsymbol{r}( \boldsymbol{c},\boldsymbol{f})|\boldsymbol{f}]. $$

The second equality holds because the masks are independent and identically distributed. For a finite variance $\mathbb{V}[\boldsymbol{r}(\boldsymbol{c},\boldsymbol{f})|\boldsymbol{f}]$, the root mean square error between the estimator and its expectation thus scales as $O(n^{-1/2})$. □

#### Acceleration by low-discrepancy sequences

When the masks are random variables, as noted in Sect. 3.1.4, we have a somewhat slow convergence of $O(n^{-1/2})$. Informally this means that to decrease the RMSE by a factor 4 we would need 16 times as many samples. The natural question arises whether we can do better by trading randomness for a more structured sampling strategy. The answer is positive, as in the context of integration (and our problem can be framed as such with respect to the counting measure), a prominent approach for speeding up convergence is the use of low-discrepancy sequences. These sequences fill up space more uniformly than random sequences. The uniformity is typically quantified using the (star) discrepancy of the sequence. Theoretically, the Koksma–Hlawka inequality [91] allows one to bound the numerical integration error, i.e., $\|\langle \boldsymbol{u}\rangle _{n}-\mathbb{E}[\langle \boldsymbol{u}\rangle _{n}| \boldsymbol{f}]\|_{2}$ in our case, by using the product of the discrepancy of the sequence and the variation of the integrand. In practice this usually translates to a convergence that can reach as high as $O(n^{-1})$ which is much better than the $O(n^{-1/2})$ convergence for the purely random case. Experimental results illustrating a boost to the convergence in the DbI setting are presented in Sect. 6.2.

## Linking denoising by inpainting to homogeneous diffusion

The simplest approaches for mask selection in the DbI framework are those, that are independent of the image that is to be filtered ($p(\boldsymbol{c}| \boldsymbol{f}) \equiv p(\boldsymbol{c})$). We consider shifted regular masks as well as randomly selected masks. They are characterized by a spatially flat expectation $\mathbb{E}[\boldsymbol{c}] = \mathrm{const}$. In the following, we briefly introduce regular masks, show how they can be used in the DbI framework and discuss the resulting filtering behavior. Then we derive relations between DbI with regular masks and homogeneous diffusion filtering in 1D. Afterwards, using random masks instead of regular masks, we empirically extend those results to the 2D setting.

### Regular masks

Regular masks are created by generating a pattern with each *r*th pixel in the *x*- and each *s*th pixel in the *y*-direction being added to the mask. We can then shift such a mask in both directions to obtain multiple masks. If we assume an $n_{x} \times n_{y}$ pixel grid, we can create such a regular mask via

45 $$ c_{i, j} = \textstyle\begin{cases} 1 & \text{if } i \bmod r = 0 \text{ and } j \bmod s = 0, \\ 0 & \text{else.} \end{cases} $$

We have *r* options of shifting this regular mask in the *x*-direction and *s* options in the *y*-direction, adding up to $n=rs$ total possible configurations. Denoting by $p \in \{0, \dots , r-1\}$ and $q \in \{0, \dots , s-1\}$ the shift in the *x*- and *y*-direction, respectively, we can write the shifted masks as

46 $$ c^{ps+q+1}_{i, j} = \textstyle\begin{cases} 1 & \text{if } i \bmod r = p \text{ and } j \bmod s = q, \\ 0 & \text{else.} \end{cases} $$

**Figure 2:**
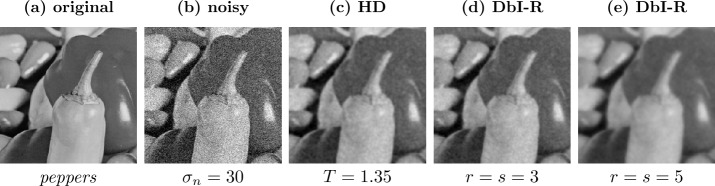
Comparison of homogeneous diffusion (HD) and denoising by inpainting with regular masks (DbI-R) on the test image *peppers* with $\sigma _{n}=30$. Figures 2(c) and 2(d) show the visual similarities of both methods. Figures 2(d) and 2(e) illustrate the influence of the expected density $\boldsymbol{1}^{\mathsf{T}}\mathbb{E}[\boldsymbol{c}]/N$ on the smoothness of the reconstruction: Fig. 2(e) was intentionally chosen with a density that is too low, resulting in too much smoothing

Clearly, the created masks are independent of the image. Furthermore, the mask density is constant over the entire image, leading to the same smoothing strength at all locations, solely determined by the total mask density, i.e., by the spacing. If $r=s$, this smoothing is equally strong in the *x*- and *y*-direction. Visually one then observes a smoothing behavior that resembles the one of homogeneous diffusion filtering (see Figs. 2(c) and 2(d)). The influence of the mask density on the smoothing strength can be observed in Figs. 2(d) and 2(e).

The similarity between the methods can not only be observed visually, but also established theoretically. Next we provide a derivation in the 1D case for regular masks relating the diffusion time of homogeneous diffusion to the mask density in DbI.

### Mathematical analysis in 1D

We consider a discrete 1D signal ***f*** and regular inpainting masks with spacing *r* and shift $p \in \{0, \dots , r-1\}$. It is known that in 1D, homogeneous diffusion inpainting and linear interpolation are equivalent. Thus, an inpainted pixel at position *i* can be described in terms of its two neighboring mask pixels. We denote the distance between the pixel *i* and its neighboring mask pixel on the left by $\ell :=|i-p| \bmod r$, which implies that for mask pixels we have $\ell =0$. Accordingly, the distance to the mask pixel on the right is given by $r-\ell $. The interpolated value at pixel *i* for mask $\ell +1$ is then

47 $$ v_{i}^{\ell +1} = \frac{r-\ell}{r} \, f_{i-\ell} + \frac{\ell}{r} \, f_{i+r- \ell}. $$

To obtain the final result, the inpaintings from the *r* shifted masks are averaged. We get

48 $$ \begin{aligned} u_{i} = \frac{1}{r} \sum _{\ell =0}^{r-1} v_{i}^{\ell +1} &= \frac{1}{r} \left (f_{i} + \sum _{\ell =1}^{r-1} \frac{r-\ell}{r} \, f_{i- \ell} + \frac{\ell}{r} \, f_{i+r-\ell}\right ) \\ &= \frac{1}{r^{2}} \left (r \, f_{i} + \sum _{\ell =1}^{r-1} \ell \left (f_{i-(r- \ell )} + f_{i+(r-\ell )}\right )\right ), \end{aligned} $$

where the last line reveals the general form of the filter in dependence of the spacing *r*. The filter is given by a hat kernel with central weight $1/r$ and width $2r-1$. In Theorem 4 we demonstrate that this kernel can be seen as a consistent discretization of $\partial _{t} u = \partial _{xx}u$. Consequently, convolution with such a kernel approximates Gaussian smoothing, which explains the visual similarity of the results in Fig. 2. Since the spacing *r* determines the size of the smoothing kernel, we explicitly see the connection between the mask density and the smoothing strength. For the special case of $r=2$, (48) yields

49 $$ u_{i} = \frac{f_{i-1} + 2 \, f_{i} + f_{i+1}}{4}, $$

which is exactly a single step of an explicit scheme for homogeneous diffusion with step size $T = \frac{1}{4}$ and initial signal ***f*** (assuming grid size $h=1$). If we reformulate (48) in a way that resembles an explicit scheme for homogeneous diffusion, we can derive a general connection between the spacing *r* (and thus the density) of denoising by inpainting with regular masks and the time step size of such an explicit scheme, which we state in Theorem 4.

#### Theorem 4

(Connection between Mask Density and Diffusion Time)

*Given the* *r* *shifted regular inpainting masks in* 1*D*, *each of density* $d = 1/r$, *denoising by inpainting approximates explicit homogeneous diffusion at time*

50 $$ \boxed{T = \frac{1-d^{2}}{12 d^{2}}.} $$

#### Proof

In (48) we derived the general form of the filter corresponding to denoising by inpainting with regular masks of spacing *r* as

51 $$ u_{i} = \frac{1}{r^{2}} \left (r \, f_{i} + \sum _{\ell =1}^{r-1} \ell \left (f_{i-(r-\ell )} + f_{i+(r-\ell )}\right ) \right ). $$

We can rewrite this in the following manner:

52 $$ \begin{aligned} u_{i} &= \frac{1}{r^{2}} \left (r \, f_{i} + \sum _{\ell =1}^{r-1} \ell \left (f_{i-(r-\ell )} + f_{i+(r-\ell )}\right ) \right ) \\ &= \frac{1}{r^{2}} \left ( r^{2} \, f_{i} - 2 \sum _{\ell =1}^{r-1} \ell \, f_{i} + \sum _{\ell =1}^{r-1} \ell \left (f_{i-(r-\ell )} + f_{i+(r- \ell )} \right ) \right ) \\ &= f_{i} + \frac{1}{r^{2}} \sum _{\ell =1}^{r-1} \ell \left (f_{i-(r- \ell )} - 2 \, f_{i} + f_{i+(r-\ell )} \right ), \end{aligned} $$

where we have used that $\sum _{\ell =1}^{r-1} \ell = \frac{(r-1)r}{2}$. Then we may write

53 $$ \begin{aligned} u_{i} - f_{i} &= \frac{1}{r^{2}} \sum _{\ell =1}^{r-1} \ell \left ( f_{i-(r-\ell )} - 2 \, f_{i} + f_{i+(r-\ell )} \right ) \\ &= \frac{1}{r^{2}} \sum _{\ell =1}^{r-1} \ell (r-\ell )^{2} \, \frac{ f_{i-(r-\ell )} - 2 \, f_{i} + f_{i+(r-\ell )}}{(r-\ell )^{2}} \\ &= \sum _{\ell =1}^{r-1} \frac{\ell (r-\ell )^{2}}{r^{2}} \, \frac{ f_{i-(r-\ell )} - 2 \, f_{i} + f_{i+(r-\ell )}}{(r-\ell )^{2}}. \end{aligned} $$

By approximating $f_{i \pm (r-\ell )}$ via a Taylor expansion and using the sampling distance *h*, we can derive the time step size as

54 $$ \begin{aligned} u_{i} - f_{i} &= \sum _{\ell =1}^{r-1} \frac{\ell (r-\ell )^{2}}{r^{2}} \, \frac{ f_{i-(r-\ell )} - 2 \, f_{i} + f_{i+(r-\ell )}}{(r-\ell )^{2}} \\ &= \sum _{\ell =1}^{r-1} \left (\frac{\ell (r-\ell )^{2}}{r^{2}} \right ) \left (h^{2} \left . d_{xx} \, f \right |_{i} + \frac{h^{4} (r-\ell )^{2}}{12} \left . d_{xxxx} \, f \right |_{i} + \mathcal{O}(h^{6}) \right ) \\ &= h^{2} \sum _{\ell =1}^{r-1} \left ( \frac{\ell (r-\ell )^{2}}{r^{2}} \right ) \left ( \left . d_{xx} \, f \right |_{i} + \mathcal{O}(h^{2}) \right ) \\ &\approx h^{2} \sum _{\ell =1}^{r-1} \left ( \frac{\ell (r-\ell )^{2}}{r^{2}} \right ) \left . d_{xx} \, f \right |_{i}. \end{aligned} $$

We end up with an approximation of an explicit scheme with time step size

55 $$ T = h^{2} \sum _{\ell =1}^{r-1} \frac{\ell (r-\ell )^{2}}{r^{2}} = \frac{h^{2} (r^{2}-1)}{12}. $$

Using that the density is the inverse of the grid spacing and setting $h=1$, we derive the final relation between *T* and the density *d*, given by

56 $$\begin{aligned} T = \frac{1-d^{2}}{12 d^{2}}. \end{aligned}$$

 □

### Empirical extension to 2D

To derive the relationship to the diffusion time in the 1D case we used the fact that the solution of the Laplace equation with Dirichlet boundaries is given by linear interpolation. That is, we know the closed form of the inpainting echoes in 1D. In 2D a closed form solution for those is not known, however, it may be computed numerically. Thus our goal is to establish a relationship between the diffusion time and the density numerically.

We take as a starting point the ansatz from the 1D case that the diffusion time *T* is given as $\frac{1-d^{2}}{12d^{2}}$, but generalize it to the form $T\approx \frac{1-d^{\gamma}}{\beta d^{\gamma}}$. Provided that this conjecture is correct, we only need to find the constants *β* and *γ*. Since regular masks only allow for a stepwise adaptation of the mask density, they are not well suited for generating a large number of data points at different densities. Therefore, we use uniform random masks instead, which also have a spatially flat expectation, i.e., $\mathbb{E}[\boldsymbol{c}] = \mathrm{const}$.

First, we numerically tabulate the relationship between the density and the diffusion time. That is, given a density *d* we find the diffusion time $T(d)$ which minimizes the difference between the filter matrices,

57 $$ T(d) = \underset{T\geq 0}{\operatorname*{\textrm{argmin}}} \,\|\boldsymbol{A}_{DbI}(d) - \boldsymbol{A}_{HD}(T) \|^{2}_{F}. $$

Here $\|\cdot \|_{F}$ is the Frobenius norm, and the matrices are the DbI filter matrix resulting from a probability mass function for masks with expected density *d*, and the matrix modeling homogeneous diffusion at time *T* using an implicit Euler discretization:

58 $$\begin{aligned} \boldsymbol{A}_{DbI} (d) &:=\mathbb{E}\left [\left (\boldsymbol{C}+ \left ( \boldsymbol{I}-\boldsymbol{C}\right )\boldsymbol{L}\right )^{-1}\boldsymbol{C}\right ], \quad \frac{1}{N}\boldsymbol{1}^{\mathsf{T}}\mathbb{E}[\boldsymbol{c}] = d, \end{aligned}$$

59 $$\begin{aligned} \boldsymbol{A}_{HD}(T) &:=\left (\boldsymbol{I}+T \boldsymbol{L}\right )^{-1}. \end{aligned}$$

**Figure 3:**
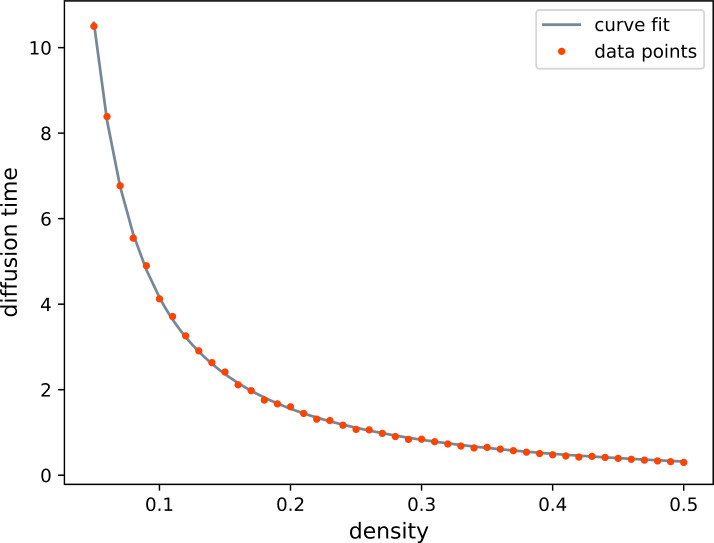
The fit based on the ansatz $\frac{1-d^{\gamma}}{\beta d^{\gamma}}$ with $\beta =4.58$, $\gamma = 1.3$ and the tabulated correspondence between density and diffusion time. The results are obtained with denoising by inpainting with uniform random masks and an implicit scheme for homogeneous diffusion. They show that also in 2D our ansatz captures the relation between mask density and diffusion time very accurately

We estimate $\boldsymbol{A}_{DbI}$ using 1024 sampled masks. Then, having the relationship $d \mapsto T(d)$ we find that $T(d) \approx \frac{1-d^{\gamma}}{\beta d^{\gamma}}$ for $\beta = 4.58$, $\gamma = 1.3$, which is illustrated in Fig. 3. Note the high quality of the data fit, which confirms the accuracy of the derived relation.

## Spatial optimization for denoising by inpainting

As we have seen in Sect. 4, the use of nonadaptive masks restricts the DbI framework, as it entails a nonadaptive smoothing behavior. Furthermore, our results from Sect. 3.1 emphasize the importance of spatial optimization in the context of image denoising. In [10], an adaptive mask selection approach enables the framework to perform edge-preserving image filtering, although the simple homogeneous diffusion inpainting operator by itself is space-invariant. This approach thus implies a different paradigm for image denoising: *Instead of optimizing the denoising operator, one can optimize the data.* In this section, we will first present the strategy that was proposed in [10]. Then we propose an alternative, simpler approach that eventually gains its power by the application of tonal optimization.

### Densification method

Two well-known mask selection strategies from image compression are probabilistic sparsification [39] and densification [42], which build the mask in an iterative way using a top-down and a bottom-up strategy, respectively.

In probabilistic sparsification, we start with a full mask and take away the least important pixels from a number of randomly selected candidates in each iteration. To identify those pixels, we temporarily exclude all candidates from the mask and compute an inpainting. Then the candidate locations with the highest local (i.e., pixelwise) reconstruction error are added back to the mask as they are assumed to be the most important, while the others remain permanently excluded. This process is repeated until the desired mask density is reached. In probabilistic densification, the initial mask is empty and again a number of candidate pixels are selected. Given an inpainting with the current mask (in the first step some pixels have to be chosen at random) we select and add those candidates to the mask that have the highest local reconstruction error.

In the noisy setting, special care is required as the pixel selection based on the *local* reconstruction error is not reliable. The local error does not allow the algorithm to distinguish between noise and important image structures, such as edges. If a pixel contains strong noise, this creates a large local error because – just like edges – the noise cannot be reconstructed by the smooth inpainting. Introducing such a noisy pixel into the mask is not desirable. We cure this problem by judging the importance of a pixel based on its effect on the *global* reconstruction error. We do this by calculating a full inpainting for each candidate pixel. While this improves the quality of the selected mask, it drastically increases the run time.

**Figure 4:**
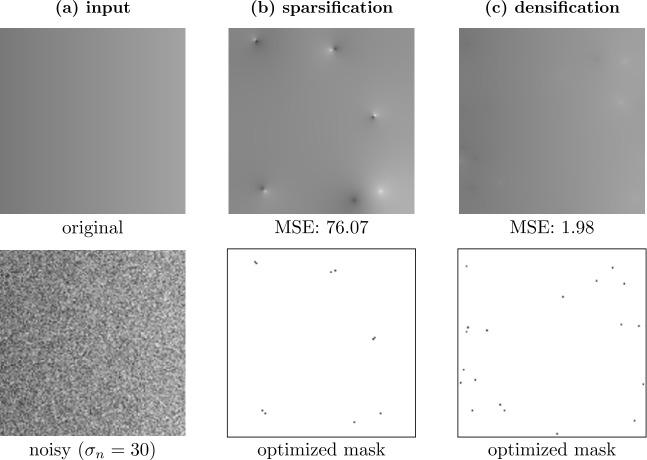
Comparison of sparsification and densification on a synthetic test image with $\sigma _{n}=30$ [10]. For both methods, the mask density *d* was optimized with a grid search with respect to the MSE. The noisy gradient image is not reconstructed adequately by sparsification, since it favors keeping noisy pixels in the first iterations due to localization. Densification does not suffer from this problem and thereby achieves a better denoised reconstruction

Even though in the noise-free setting densification and sparsification yield results of comparable quality [38], this is different when handling noisy data. For sparsification, we initially have very dense masks. If we exclude candidate pixels from such masks, the reconstructions often only differ at the locations of these pixels. Therefore, sparsification tends to keep noisy pixels in the mask, even when a global reconstruction error is computed. This problem does not occur in probabilistic densification, as for a sparse mask, the candidate pixels have a global influence. The result of this effect is illustrated in Fig. 4. Here, densification is able to select appropriate pixels that lead to an almost perfect result while sparsification fails to reconstruct the image properly.

Thus, we opt for a *probabilistic densification* algorithm based on a *global* error computation, which is described in Algorithm 1 and has been proposed in [10]. An additional advantage of this probabilistic densification method is that it does not only select pixels at useful locations (e.g., close to edges), but also implicitly avoids picking pixels that are too noisy, as they would have a negative impact on the reconstruction quality. The method can be interpreted according to the probabilistic mask generation framework from Sect. 3, and we provide the implied mask probabilities in the following proposition.

**Algorithm 1:**
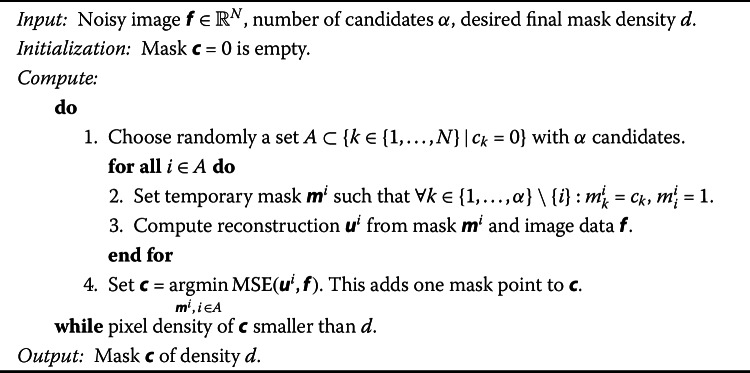
Mask densification with global error computation [10]

#### Proposition 5

(Mask Probabilities implied by the Densification Method)

*A mask* ***c*** *generated by probabilistic densification has the conditional probability density function*

60 $$ p(\boldsymbol{c}|\boldsymbol{f}) = \sum _{\sigma \in S_{m}}p_{\sigma}(\boldsymbol{c}|\boldsymbol{f}), $$

*where* $m=\|\boldsymbol{c}\|_{0}$ *is the number of mask pixels*, *the sum is taken over the group* $S_{m}$ *of permutations of the ordering of the* *m* *mask pixels*, *and* $p_{\sigma}(\boldsymbol{c}|\boldsymbol{f})$ *denotes the probability that the* *m* *mask points were introduced in the order* *σ*. *The latter is the product of the probabilities of selecting one mask pixel at each step*,

61 $$ p_{\sigma}(\boldsymbol{c}|\boldsymbol{f}) = p^{m}_{\sigma}(\boldsymbol{c}|\boldsymbol{f}) \cdots p^{1}_{ \sigma}(\boldsymbol{c}|\boldsymbol{f}). $$

*The probability of picking the* *kth mask pixel* (*according to the permutation* *σ*) *at step* *k* *has the following form*:

62 $$ p^{k}_{\sigma}(\boldsymbol{c}|\boldsymbol{f}) = \sum _{\beta =1}^{\alpha} \frac{1}{\beta} \frac{\binom{N_{eq}-1}{\beta -1} \binom{N_{gt}}{\alpha -\beta}}{\binom{N-k}{\alpha}}, $$

*where* *α* *is the number of candidates considered per step*, $N_{gt}$ *is the number of nonmask pixels at step* *k* *that would have resulted in an inpainting with a higher MSE if they were chosen instead of the* *kth mask pixel in* *σ*, *and* $N_{eq}$ *is the number of nonmask pixels that would have resulted in the same MSE*.

#### Proof

We present the proof of this result in Appendix A. □

### Acceleration via the analytic results of Belhachmi et al

As the global error computation in the previous approach requires calculating an inpainting for each candidate pixel, the run time is substantial. Therefore, we propose another approach, with the goal of a faster mask generation process. We refer to this method as the *analytic method*. It is based on the results of Belhachmi et al. [20]. They have shown that the mask density for homogeneous diffusion inpainting should be proportional to the pointwise magnitude of the Laplacian $|\boldsymbol{L}\boldsymbol{f}|$. Additionally, they suggest using the Gaussian-smoothed version $\boldsymbol{f}_{\sigma} :=\boldsymbol{K}_{\sigma} \ast \boldsymbol{f}$ of ***f*** even in the noise-free setting. Here $\boldsymbol{K}_{\sigma}$ is a discrete approximation of a Gaussian with standard deviation *σ*. This step proves even more beneficial in our setting, since we are calculating the Laplacian of noisy data, and regularizing ***f*** helps considerably for constructing a reasonable guidance image $|\boldsymbol{L}\boldsymbol{f}_{\sigma}|$.

As we require multiple different binary masks for our framework, we sample from $|\boldsymbol{L} \boldsymbol{f}_{\sigma}|$ by using a simple and fast Poisson sampling. Given a density image $\boldsymbol{d}\in [0,1]^{N}$, we can sample a mask according to it by generating a uniform random number $v_{i}\sim U[0,1]$ for each pixel *i* and then thresholding at $d_{i}$:

63 $$ c_{i} = \textstyle\begin{cases} 1 & \text{if } v_{i} \leq d_{i}, \\ 0 & \text{if } v_{i} > d_{i}.\end{cases} $$

Then the probability mass function $p_{\boldsymbol{d}}$ for sampling a mask ***c*** given the density image ***d*** is

64 $$ p_{\boldsymbol{d}}(\boldsymbol{c}) = \frac{1}{P}\prod _{i=1}^{N} (d_{i})^{c_{i}}(1-d_{i})^{1-c_{i}}, \quad P = \sum _{\boldsymbol{c}\in \{0,1\}^{N}}\prod _{i=1}^{N} (d_{i})^{c_{i}}(1-d_{i})^{1-c_{i}}. $$

**Figure 5:**
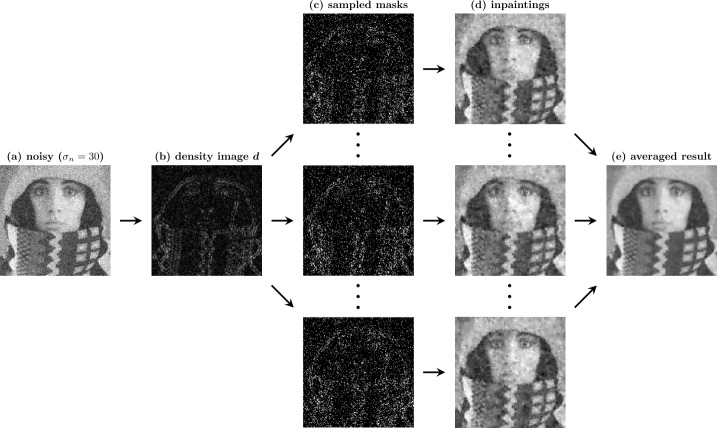
Pipeline for mask generation with the analytic method. (a) Test image *trui* with $\sigma _{n}=30$. (b) Target image (without postsmoothing) from which masks are sampled. (c) Three examples of Poisson-sampled masks. (d) Corresponding homogeneous diffusion inpaintings. (e) Averaged inpaintings (from 32 masks), final denoising result

By construction the mask would have an expected density equal to the mean value of ***d***. In our approach we set the per pixel probabilities to

65 $$ \boldsymbol{d} = \min \left \{C|\boldsymbol{L}\boldsymbol{f}_{\sigma}|,\boldsymbol{1}\right \}, $$

where the minima are taken pointwise, and *C* is a constant chosen such that the mean value of ***d*** is equal to the desired mask density. Figure 5 shows the pipeline for mask generation with this method. One can observe in Fig. 5(b) that ***d*** is strongly affected by the noise despite the presmoothing. This is because we calculate second-order derivatives that are even more sensitive to noise. When sampling from this image the mask is drawn towards noisy pixels. To counteract this, we propose to perform an additional outer smoothing of the probability image ***d***, after the absolute value of the Laplacian is taken, thus modifying it to

66 $$ \boldsymbol{d} = \min \left \{C \left (\boldsymbol{K}_{\rho} \ast |\boldsymbol{L}\boldsymbol{f}_{ \sigma}|\right ), \boldsymbol{1}\right \}, $$

with a postsmoothing parameter *ρ*. Our proposed selection strategy offers an instant generation of adaptive masks, in a sense that it does not require the calculation of any inpainting. Furthermore, it provides a transparent formulation of the mask PMF (see (64)) and as such exhibits a specifically simple interpretation in the context of our probabilistic framework in Sect. 3.1. On the other hand, contrary to probabilistic densification it does not have a mechanism to avoid noisy mask pixels. To obtain the best possible results, the presmoothing parameter *σ*, the postsmoothing parameter *ρ*, and the desired mask density have to be optimized depending on the image content and the noise level.

Note that Belhachmi et al. [20] apply Floyd–Steinberg dithering [92], which includes an error diffusion in the binarization process. This strategy can be equipped with a random component in order to generate multiple masks, which makes it an alternative to Poisson sampling for us. We have tested both methods and found that there is no advantage in using Floyd–Steinberg dithering. Thus, we opt for the simple Poisson sampling. Nonetheless, we give the mask probabilities for sampling with error diffusion methods in Appendix B.

## Experiments

In this section, we present our experiments. They evaluate our theories and compare the different DbI strategies in practice. Firstly, we confirm the accuracy of the 1D relation that we derived for DbI with regular mask in Sect. 4.2. We also display the corresponding results in 2D. Next, we show that the theoretical convergence estimates from Sect. 3.1.4 also hold in practice and evaluate the gain through low-discrepancy-based sampling (see Sect. 3.1.5). Furthermore, we assess the spatial and tonal mask optimization approaches. To this end, we compare DbI to PDE-based methods of similar structural complexity. Aside from homogeneous diffusion, we choose linear space-variant diffusion and nonlinear diffusion as representatives of methods that are based on operator optimization. Lastly, we consider the denoising by biharmonic inpainting to further investigate the question of data optimization vs. operator optimization.

### Relation between DbI and homogeneous diffusion

**Figure 6:**
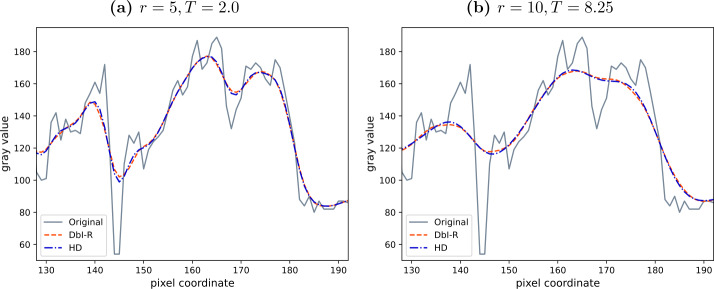
Comparison of denoising by inpainting with shifted regular masks (DbI-R) and homogeneous diffusion (HD) on a one-dimensional signal (128th row of the test image *peppers*). We display a section from the original signal and filtered versions obtained with denoising by inpainting with regular masks of spacing *r* and homogeneous diffusion filtering with diffusion time *T*, calculated according to Theorem 4. We see that both filters lead to very similar results, confirming that the approximation from the theorem is indeed realistic

In Sect. 4.2 we derived a relation between the mask density *d* and the diffusion time *T*, given by $T = (1-d^{2})/(12 d^{2})$. To confirm that this relation allows for a good estimate of the diffusion time in practice, we perform an experiment on a 1D signal, which is generated by extracting the 128th row of the *peppers* test image. Homogeneous diffusion is implemented using explicit Euler and the spatial discretization from (49) with the number of iterations chosen such that the desired diffusion time *T* is reached. The result in Fig. 6 demonstrates that the diffusion time obtained via Theorem 4 is a good approximation.

**Figure 7:**
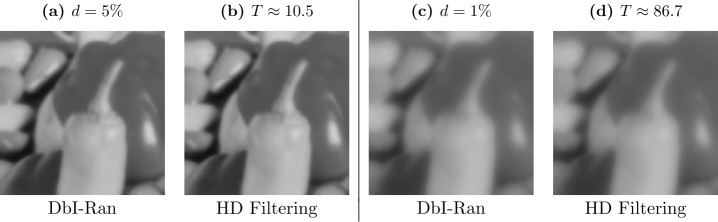
Comparison of denoising by inpainting with 1024 random masks (DbI-Ran) and homogeneous diffusion (HD) on the test image *peppers*. The diffusion times *T* corresponding to the mask densities *d* are calculated according to the result from Sect. 4.3. The MSE between (a) and (b) is 0.61 and the MSE between (c) and (d) is 6.37. This shows that the empirically derived relation is accurate, even for longer diffusion times

In Sect. 4.3 we extended this relation to 2D, yielding $T = (1-d^{\gamma})/(\beta d^{\gamma})$ with $\beta =4.58$ and $\gamma = 1.3$. To confirm this, we now consider the 2D *peppers* test image. We perform denoising by inpainting with 1024 randomly selected masks, as well as homogeneous diffusion filtering with the diffusion time calculated according to the above relation and compare the results. The experiments in Fig. 7 visually and qualitatively confirm the accuracy of the relation in 2D.

### Convergence

**Figure 8:**
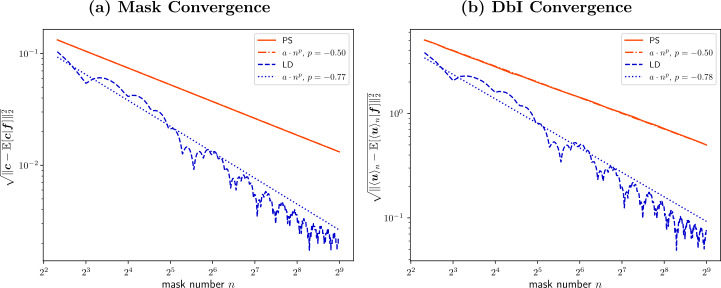
Convergence results for denoising by inpainting with the analytic method with Poisson sampling (PS) vs. low-discrepancy-based sampling (LD): (a) the convergence of the masks; (b) the convergence of the DbI result

As we have shown in Sect. 3.1.4 the estimator converges to its expectation at a rate of $O(n^{-1/2})$ with respect to the RMSE. In Sect. 3.1.5 we introduced the idea of using low-discrepancy sequences. Theoretically, they should lead to much faster convergence, thus here we test whether this also holds in practice. In the experiments we again use the $256\times 256$ test image *peppers*. We use two sampling strategies for the masks, whose sample means $\boldsymbol{c} = \frac{1}{n}\sum _{\ell =1}^{n}\boldsymbol{c}^{\ell}$ converge to the same expectation $\mathbb{E}[\boldsymbol{c}|\boldsymbol{f}]$. As a representative of a low-discrepancy sequence we use the R2 sequence [93] to create a sampling threshold in each pixel (see [93] for details). This leads to a more regular sampling pattern compared to using a purely random threshold. To make the experiment relevant to realistic scenarios, we use the analytic mask selection method from Sect. 5.2. We first test the mask convergence. To this end, we create $2^{16} = 65{,}536$ masks via Poisson sampling and consider their average as converged to the expectation $\mathbb{E}[\boldsymbol{c}|\boldsymbol{f}]$. Then we sample masks with both sampling strategies and observe how the RMSE between sample mean and expectation evolves with *n*. Of course, we are more interested in the convergence of the DbI result $\langle \boldsymbol{u}\rangle _{n}$. Therefore, following a similar approach as for the masks, we create an individual “converged” DbI result for the two sampling methods, and again consider the RMSE between $\langle \boldsymbol{u}\rangle _{n}$ and the respective reference images. Figure 8 shows that the simple Poisson sampling leads to a convergence rate of $O(n^{-1/2})$ for the masks as well as for the DbI result, which is perfectly in line with the theory from Sect. 3.1.4. Through low-discrepancy sampling this rate approaches $O(n^{-1})$. By fitting a curve through the data, we get a convergence rate of $O(n^{-0.77})$ for the masks and $O(n^{-0.78})$ for the DbI result. The $O(n^{-1})$ estimate is typically achieved for low dimensions, so the difference of our results can be explained by the high dimensionality of our sampling problem. The experiments confirm that the sampling strategy based on low-discrepancy sequences is indeed able to improve the convergence in practice.

### Data optimization for denoising by inpainting

In the next step, we investigate the edge-preserving filtering behavior achieved by the use of adaptive masks. We first test the two spatial optimization methods and compare the results to classical diffusion models. We show that DbI can yield results comparable to certain space-variant diffusion methods. Then we discuss the effect of tonal optimization in the DbI setting. It should be noted that these experiments are meant to provide an illustration of the mask optimization strategies and not to achieve the best denoising quality. As we have shown, these strategies can be applied in a more general setting than DbI with homogeneous diffusion inpainting. They are valid for the general probabilistic framework from Sect. 3.1, and as such they also extend to more complex operators (including nonlinear ones).

We perform experiments on the three standard test images *trui*, *peppers*, and *walter* with a resolution of $256\times 256$, that are corrupted with additive Gaussian noise with standard deviations $\sigma _{n} \in \{10, 20, 30\}$ that we do not clip. To ensure a fair comparison, we optimize the mask density and if required the pre- and postsmoothing parameter for the denoising by inpainting methods with respect to the MSE to the original image. We do this individually for each image and for each noise level using a grid search. In practice, these parameters need to be adapted to the noise level and the image content. We create 32 masks with each of the mask selection methods, except for the regular masks where the number is determined by the spacing and thus by the density. For the proposed probabilistic densification algorithm we set the number of candidate pixels per iteration to 16.

#### Spatial optimization

Firstly, we investigate the different spatial selection strategies proposed in Sect. 5 and compare the denoising results with the standard diffusion methods presented in Sect. 2.1. For the diffusion methods, which we discretize with an explicit scheme, we optimize the stopping time and if required the contrast parameter of the Charbonnier diffusivity [88].

**Figure 9:**
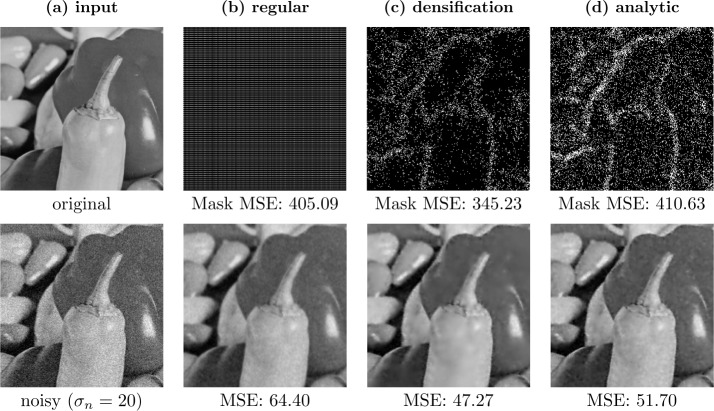
Results for denoising by inpainting with 32 masks (six masks for the regular mask method) for the different spatial optimization methods on the test image *peppers* with $\sigma _{n}=20$: (top) (a) original image, (b)–(d) one representative out of all the masks for every method. The MSE is computed at mask pixels; (bottom) (a) noisy image, (b)–(d) denoising by inpainting results with optimized parameters and the MSE in the entire image. We see that our analytic method and the densification method adapt the mask point locations to the structure of the image. Densification additionally avoids choosing noisy mask pixels, leading to a smaller error in the mask pixels and eventually to a better reconstruction

As can be seen in Table 1, inpainting with regular masks leads to unsatisfying results, slightly worse than those obtained with homogeneous diffusion filtering. This is expected given the connections derived in Sect. 4.1. Note that the stopping time in homogeneous diffusion filtering can be tuned continuously, while the spacing of the regular mask can only be adapted in integer steps. The analytic method based on Poisson sampling of the smoothed Laplacian magnitude improves the results, especially at lower noise levels. Figure 9(c) shows how the mask pixels accumulate around important image structures, enabling an edge-preserving filtering behavior. The densification method is able to further improve those results. The reason for this improvement can be seen in Fig. 9(d). On top of selecting pixels at reasonable positions, the error in the mask is reduced drastically in comparison to the analytic method, because densification implicitly avoids noisy pixels. The adaptive mask selection strategies enable the denoising by inpainting method to produce results that are comparable to linear space-variant diffusion filtering. However, it cannot reach the quality of nonlinear diffusion. This is not surprising, as a feedback mechanism throughout the inpainting process is missing. Nonetheless, the results reveal that proper data optimization enables DbI to compete with methods that optimize the operator, if they are of comparable complexity.

**Table 1:** Results (MSE) for denoising by inpainting with regular masks, the densification method and the analytic method with 32 masks (fewer masks for the regular mask method). Comparison to classical diffusion-based denoising methods

		trui			peppers			walter		
	noise level $\sigma _{n}$	10	20	30	10	20	30	10	20	30
DbI	regular	27.30	57.29	86.46	35.31	64.40	91.79	22.63	50.13	79.16
	densification	**19.34**	**42.72**	**68.01**	**24.36**	**47.27**	**69.89**	**13.40**	**29.65**	**47.65**
	analytic	21.49	49.71	79.79	25.14	51.70	79.91	16.41	37.83	62.08
Diff	homogeneous	24.12	50.18	76.12	32.16	59.77	84.58	19.65	42.76	66.87
	lin. space-var.	17.89	42.62	69.57	24.03	47.47	72.67	13.31	32.30	55.37
	nonlinear	**16.21**	**34.99**	**54.66**	**22.63**	**40.48**	**57.54**	**11.89**	**25.31**	**39.49**

Although qualitatively the densification approach is better than the analytic method, its required run time is orders of magnitude larger, and this only gets worse for images of higher resolution. Due to the required number of inpaintings, the densification method takes about an hour to create a single mask with 10% density for our $256\times 256$ pixel test images. In contrast, the analytic and the regular approaches allow instant mask generation in approximately a millisecond. Thus, the analytic method yields a reasonable spatial mask pixel distribution in a very short time and clearly has potential, if the error in the mask pixels can be reduced. We show next that this can be achieved by complementing the mask selection strategies with tonal optimization.

#### Tonal optimization

As mentioned in Sect. 3.1.1, tonal optimization leads to an MMSE estimate that is approximating instead of interpolating. If one assumes that mask pixels are erroneous due to the noise, this is certainly a desirable behavior. We will evaluate its effect in the following. To this end, we apply tonal optimization to the masks obtained by each of our spatial optimization methods. We optimize the tonal values for each individual mask, before once again averaging the respective inpaintings to obtain the final denoised result.

The results in Table 2 reveal that the methods that do not consider the noise in the selection process get the greatest boost in performance. This confirms the conjecture that tonal optimization is able to mitigate the negative effect of noisy mask pixels selection. We also observe that tonal optimization decreases the error in the mask pixels for those methods. In Fig. 9, the MSE at mask locations decreases from 405.09 to 271.80 for the regular mask, and from 410.63 to 306.62 for the analytic method. For probabilistic densification, tonal optimization barely changes the final results, as well as the mask MSE (which even increases slightly from 345.23 to 356.12 in the example).

**Table 2:** Results (MSE) for denoising by inpainting with regular masks, the densification method and the analytic method with 32 masks (less masks for the regular mask method) including tonal optimization. Comparison to classical diffusion-based denoising methods

		trui			peppers			walter		
	noise level $\sigma _{n}$	10	20	30	10	20	30	10	20	30
DbI	regular	22.32	48.77	76.06	32.65	60.92	87.05	16.36	39.54	64.45
	densification	18.46	41.56	67.72	24.42	47.28	70.21	12.35	28.13	45.92
	analytic	**17.24**	**39.49**	**63.17**	**23.68**	**46.43**	**68.55**	**12.08**	**27.66**	**45.36**
Diff	homogeneous	24.12	50.18	76.12	32.16	59.77	84.58	19.65	42.76	66.87
	lin. space-var.	17.89	42.62	69.57	24.03	47.47	72.67	13.31	32.30	55.37
	nonlinear	**16.21**	**34.99**	**54.66**	**22.63**	**40.48**	**57.54**	**11.89**	**25.31**	**39.49**

**Figure 10:**
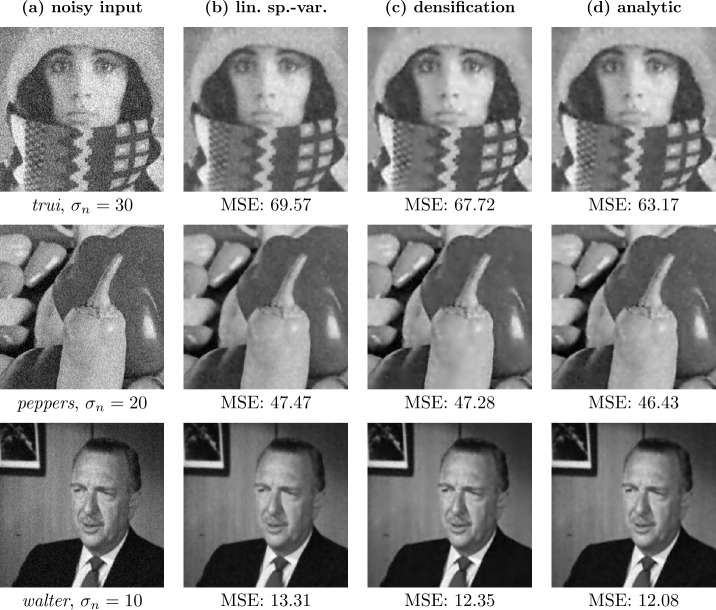
Visual comparison of linear space-variant diffusion and denoising by inpainting with the densification method and the analytic method on three test images with noise. Both DbI methods are using tonal optimization

We see that tonal optimization enables the analytic method to produce results of quality comparable to those of the densification method, and of better quality than space-variant diffusion. Although the tonal optimization step takes some additional seconds, the analytic method is still orders of magnitude faster than the densification method. Figure 10 shows a selection of resulting images comparing the two adaptive mask selection methods with tonal optimization and linear space-variant diffusion, as the diffusion method that leads to the most similar results.

### Denoising by biharmonic inpainting

Our previous results reveal that optimizing the data instead of the operator constitutes an interesting alternative for image denoising. To further substantiate this idea, we now adapt the inpainting operator within the DbI framework. We consider biharmonic inpainting as a representative of a higher-order polyharmonic operator.

It has been shown that the biharmonic operator can have quality advantages over homogeneous diffusion (i.e., the harmonic operator) in classical sparse inpainting [18, 36, 49]. Biharmonic inpainting is given by the PDE

67 $$ \bigl(c(\boldsymbol{x}) + (1 - c(\boldsymbol{x})) \Delta ^{2} \bigr)u(\boldsymbol{x}) = c( \boldsymbol{x})f(\boldsymbol{x}) \quad \text{for } \boldsymbol{x} \in \Omega , $$

with $\Delta ^{2} u = \partial _{xxxx} u + 2 \partial _{xxyy} u + \partial _{yyyy} u$ and reflecting boundary conditions $\partial _{\boldsymbol{n}}u(\boldsymbol{x}) = 0$ and $\partial _{\boldsymbol{n}}\Delta u (\boldsymbol{x}) = 0$ for $\boldsymbol{x}\in \partial \Omega $. It can be derived from the following variational formulation (analogously to (12)):

68 $$ \min _{u}\int _{\Omega}(\Delta u(\boldsymbol{x}))^{2}\,d\boldsymbol{x}, \textrm{ such that } u(\boldsymbol{x}) = f(\boldsymbol{x}) \textrm{ for } \boldsymbol{x} \in K. $$

This shows that biharmonic inpainting penalizes second-order derivatives. Biharmonic inpainting does not suffer from the typical singularities at mask points that homogeneous diffusion inpainting produces. On the other hand it can produce over- and undershoots, since it does not guarantee a maximum–minimum principle. We evaluate the potential of biharmonic inpainting for denoising by comparing it to homogeneous diffusion inpainting. To ensure that the results reflect the quality of the operators, we first perform the experiment on fully random masks.

Our results in Table 3 show that biharmonic inpainting does lead to an improvement, and it is largest at low noise levels. This is to be expected, as the method is not as radical as homogeneous diffusion inpainting, since it penalizes second- instead of first-degree derivatives. However, already tonal optimization as a first data optimization step neutralizes this advantage and the two methods perform similarly. These results support our reasoning that data optimization plays a significant role for the denoising abilities of our framework, being more important than the use of more complex, higher-order models. Further experiments on spatially optimized masks (see Table 4) confirm our findings, and even shift the advantage towards homogeneous diffusion inpainting. When comparing to previous results from classical sparse image inpainting, one has to consider that the singularities, that homogeneous diffusion inpainting suffers from, are suppressed by the averaging in the DbI framework. Thus, this disadvantage of homogeneous diffusion inpainting does not come into play in our scenario. Lastly, one should keep in mind that biharmonic inpainting leads to a higher condition number of the inpainting matrix, and consequently each inpainting is numerically more burdensome and less efficient.

**Table 3:** Results (MSE) for denoising by inpainting with 32 random masks using homogeneous diffusion (HD) and biharmonic (BI) inpainting, without and with tonal optimization (TO)

	trui			peppers			walter		
noise level $\sigma _{n}$	10	20	30	10	20	30	10	20	30
HD, without TO	30.53	65.51	100.51	36.01	71.20	104.21	26.93	60.21	94.37
BI, without TO	**24.23**	**56.37**	**93.51**	**33.28**	**66.61**	**102.49**	**19.16**	**48.19**	**82.92**
HD, with TO	23.10	49.83	**76.57**	**31.98**	**59.75**	**85.28**	18.09	41.79	66.40
BI, with TO	**22.21**	**49.74**	77.25	33.27	61.84	87.92	**16.52**	**39.53**	**65.03**

**Table 4:** Results (MSE) for denoising by inpainting with 32 masks obtained with the analytic method using homogeneous diffusion (HD) and biharmonic (BI) inpainting, without and with tonal optimization (TO)

	trui			peppers			walter		
noise level $\sigma _{n}$	10	20	30	10	20	30	10	20	30
HD, without TO	21.49	49.71	**79.79**	**25.14**	**51.70**	**79.91**	16.41	37.83	**62.08**
BI, without TO	**19.01**	**47.47**	82.39	25.83	55.42	90.28	**14.16**	**37.15**	68.25
HD, with TO	17.24	**39.49**	**63.17**	**23.68**	**46.43**	**68.55**	12.08	27.66	**45.36**
BI, with TO	**17.18**	40.45	66.13	25.35	49.27	72.68	**11.74**	**27.22**	45.70

## Conclusions

Our work is the first that links the tasks of PDE-based image inpainting and denoising in a systematic way, by providing an explicit connection between homogeneous diffusion inpainting and denoising through a relation between the diffusion time and the mask density. Our *denoising by inpainting (DbI)* framework achieves denoising by averaging inpainting results with different sparse masks of the same density. It constitutes a means to investigate the connections between PDE-based denoising and inpainting and allows us to evaluate the denoising potential of PDE-based inpainting methods. We have established a probabilistic theory with convergence estimates for the framework, and have extended it to a deterministic version by the use of low-discrepancy sequences. We have further shown that this framework computes an approximation to an MMSE estimate. For nonadaptive masks, we have linked the framework to classical diffusion via a one-to-one relationship between the mask density and the diffusion time. We have demonstrated that a simple operator can exhibit space-variant filtering behavior, when supplemented with adaptive data selection strategies. Experiments with a higher-order inpainting operator, which can be more powerful than homogeneous diffusion inpainting [18, 36, 49], have underlined the importance of choosing appropriate data over more complex operators. For data optimization specific to denoising by inpainting, we have presented two distinct, fundamental strategies. The densification method from our conference paper [10] aims at finding pixels that represent the data well. Thereby, it implicitly avoids the selection of noisy mask pixels during spatial optimization. On the contrary, we have proposed a new approach, where the selection of noisy pixels is tolerated in the spatial optimization but is compensated for by the tonal optimization.

Our work constitutes an unconventional, new viewpoint on image denoising. By using a simple inpainting operator but focusing on adequate data selection, we *shift the priority from optimizing the filter model to optimizing the considered data.* Moreover, our densification strategy allows us to find the most trustworthy pixels in the data. This shows that *simple filter operators such as homogeneous diffusion can give deep insights into data*. Last but not least, we have seen that the filling-in effect is not only useful in variational optic flow models and in PDE-based inpainting, but also in denoising. This emphasizes its fundamental role in digital image analysis, which is in full agreement with classical results from biological vision [94].

While our focus in the present paper is on gaining fundamental insights into the potential of inpainting ideas for denoising, our future work will deal with various modifications to make these ideas also applicable to more recent denoising methods. To this end, we are going to consider more sophisticated inpainting operators [49] and data selection strategies [95], including neural ones [96], and the incorporation of more advanced types of data [97]. Such future work should also extend our theory to, e.g., space-variant and nonlinear operators.

## Data Availability

The datasets used and/or analyzed during the current study are available from the corresponding author on reasonable request.

## Appendix A: Proof of Proposition 5

We derive the expression for the stated probability in Proposition 5 for step $k+1$ here. At the beginning of step $k+1$, Algorithm 1 has already inserted *k* mask pixels yielding the mask $\boldsymbol{c}^{k}$. At the end of step $k+1$, we want to have inserted a new mask pixel that is not in $\boldsymbol{c}^{k}$. Consequently, we can select a pixel only from the set $\mathcal{I}^{k}$ of remaining empty mask pixel locations, with $|\mathcal{I}^{k}|=N-k$. The algorithm samples a set $\mathcal{X}$ of *α* distinct candidates from $\mathcal{I}^{k}$ uniformly at random (there are $C^{N-k}_{\alpha}$ different ways to do so):

69 $$ \mathcal{X} = \{X_{1}, \ldots , X_{\alpha }\in \mathcal{I}^{k} \,:\, X_{i} \ne X_{j} \text{ for } i\ne j\}. $$

Then one chooses the candidate $X^{*}\in \mathcal{X}$ with lowest reconstruction error (with respect to the noisy image ***f***):

70 $$ X^{*}\in \mathcal{X}^{*} = \underset{X\in \mathcal{X}}{\operatorname*{\textrm{argmin}}}\,E^{k}(X), \quad E^{k}(X) :=\|\boldsymbol{r}(\boldsymbol{c}^{k}+\boldsymbol{e}_{X}, \boldsymbol{f})- \boldsymbol{f}\|^{2}_{2}, $$

where $\boldsymbol{e}_{X}\in \mathbb{R}^{N}$ is the zero vector modified with a one at the location corresponding to mask point *X*. The minimizer does not have to be unique; in fact the set of minimizers

71 $$ \mathcal{X}^{*} = \{X_{i}\in \mathcal{X}\,:\,E^{k}(X_{i}) = \min _{X \in \mathcal{X}}E^{k}(X)\} $$

may have more than one element ($|\mathcal{X}^{*}|>1$) in which case we choose $X^{*}$ uniformly at random from $\mathcal{X}^{*}$ with probability $\frac{1}{|\mathcal{X}^{*}|}$. This completes step $k+1$, now with a specific $X^{*} = x^{*}$ and corresponding mask $\boldsymbol{c}^{k+1} = \boldsymbol{c}^{k} + \boldsymbol{e}_{x^{*}}$. If the desired number of mask points have been achieved the algorithm ends, otherwise one proceeds to step $k+2$ in the exact same manner.

After we have inserted mask pixel $x^{*}\in \mathcal{I}^{k}$, we want to be able to compute the probability $\Pr (X^{*} = x^{*})$ of this occurring. This is equal to the probability of $x^{*}$ having been selected as a candidate,

72 $$ \Pr (x^{*}\in \mathcal{X}) = C^{N-k-1}_{\alpha -1}/C^{N-k}_{\alpha} = \frac{\alpha}{N-k}, $$

multiplied by the probability $\Pr (x^{*}\in \mathcal{X}^{*}\,|\,x^{*}\in \mathcal{X})$ that $x^{*}$ ends up in $\mathcal{X}^{*}$, which is in turn multiplied by the probability $\Pr (X^{*}=x^{*}\,|\,x^{*}\in \mathcal{X}^{*}) = \frac{1}{|\mathcal{X}^{*}|}$ of having picked $x^{*}$ from $\mathcal{X}^{*}$ uniformly at random. We thus have the following chain of conditional probabilities:

73 $$\begin{aligned} \begin{aligned} \Pr (X^{*}=x^{*}) &= \Pr (X^{*}=x^{*}\,|\,x^{*}\in \mathcal{X}^{*}) \Pr (x^{*}\in \mathcal{X}^{*}) \\ &= \Pr (X^{*}=x^{*}\,|\,x^{*}\in \mathcal{X}^{*}) \Pr (x^{*}\in \mathcal{X}^{*}\,|\,x^{*}\in \mathcal{X})\Pr (x^{*}\in \mathcal{X}) \\ &= \frac{1}{|\mathcal{X}^{*}|}\Pr (x^{*}\in \mathcal{X}^{*}\,|\,x^{*} \in \mathcal{X}) \frac{\alpha}{N-k}. \end{aligned} \end{aligned}$$

We can write the terms involving $\mathcal{X}^{*}$ in the following manner:

74 $$ \frac{1}{|\mathcal{X}^{*}|}\Pr (x^{*}\in \mathcal{X}^{*}\,|\,x^{*} \in \mathcal{X}) = \sum _{\beta =1}^{\alpha}\frac{1}{\beta} \Pr (x^{*} \in \mathcal{X}^{*}\land |\mathcal{X}^{*}|=\beta \,|\,x^{*}\in \mathcal{X}). $$

The probability on the right-hand side can be rewritten as requiring *β* of the candidates to have energy equal to $E^{k}(x^{*})$ and the remaining $\alpha -\beta $ having a strictly larger energy:

75 $$ \begin{aligned} &\Pr (x^{*}\in \mathcal{X}^{*}\land |\mathcal{X}^{*}|= \beta \,|\,x^{*}\in \mathcal{X}) \\ &= \Pr \left (\left (\bigwedge _{i=1}^{\beta}E^{k}(X_{i})=E^{k}(x^{*}) \right ) \land \left ( \bigwedge _{j=\beta +1}^{\alpha} E^{k}(X_{j})>E^{k}(x^{*}) \right )\, \Bigg|\,x^{*}\in \mathcal{X}\right ). \end{aligned} $$

To compute the above probabilities, we would need to know the total number of pixels from $\mathcal{I}^{k}$ with energy equal to $E^{k}(x^{*})$,

76 $$ N_{eq} :=|\{x\in \mathcal{I}^{k}\,:\,E^{k}(x)= E^{k}(x^{*})\}|, $$

and the total number of pixels from $\mathcal{I}^{k}$ having a strictly higher energy,

77 $$ N_{gt} :=|\{x\in \mathcal{I}^{k}\,:\,E^{k}(x)> E^{k}(x^{*})\}|. $$

From the requirement $|\mathcal{X}^{*}|=\beta $, it follows that we need to choose *β* pixels that have energy equal to $E^{k}(x^{*})$. However, $x^{*}\in \mathcal{X}$ so $E^{k}(X)=E^{k}(x^{*})$ with probability 1 for at least one candidate $X=x^{*}$. Then $\beta -1$ elements $X_{i}$ remain to be selected from $N_{eq}-1$ locations, the total number of possibilities being $C^{N_{eq}-1}_{\beta -1}$. Finally, the remaining $\alpha -\beta $ candidates must be selected from $N_{gt}$ locations, resulting in $C^{N_{gt}}_{\alpha -\beta}$ options. Using this we can compute the probability

78 $$ \frac{1}{|\mathcal{X}^{*}|}\Pr (x^{*}\in \mathcal{X}^{*}\,|\,x^{*} \in \mathcal{X}) = \sum _{\beta =1}^{\alpha}\frac{1}{\beta} \frac{C^{N_{eq}-1}_{\beta -1}C^{N_{gt}}_{\alpha -\beta}}{C^{N-k-1}_{\alpha -1}}. $$

Ultimately, we get the following probability for step $k+1$:

79 $$ \Pr (X^{*}=x^{*}) = \frac{\alpha}{N-k}\sum _{\beta =1}^{\alpha} \frac{1}{\beta} \frac{C^{N_{eq}-1}_{\beta -1}C^{N_{gt}}_{\alpha -\beta}}{C^{N-k-1}_{\alpha -1}} = \sum _{\beta =1}^{\alpha}\frac{1}{\beta} \frac{C^{N_{eq}-1}_{\beta -1}C^{N_{gt}}_{\alpha -\beta}}{C^{N-k}_{\alpha}}. $$

Through the probabilistic densification procedure, the exact same mask ***c***, with $\|\boldsymbol{c}\|_{0}$ mask pixels, can be constructed in $\|\boldsymbol{c}\|_{0}!$ different ways (the same set of mask pixels being introduced in all possible orders). That is, we get the probability mass function $p_{\sigma}(\boldsymbol{c}|\boldsymbol{f})$ over masks that also retain the order of insertion of their mask pixels (e.g., we can modify ***c*** by setting entries equal to one, to be equal to *k* – the step in which those were inserted). To get the usual probability mass function over binary masks we need to sum up the above probabilities over all $\|\boldsymbol{c}\|_{0}!$ permutations of point insertion orders. The main issue for practicality is that $N_{eq}$ and $N_{gt}$ must be known, which would require evaluating all possible $|\mathcal{I}^{k}|=N-k$ inpaintings for a single step. Nevertheless, Monte Carlo can be used to estimate the probabilities.

## Appendix B: Probability for error diffusion masks

Error diffusion half-toning (e.g., Floyd–Steinberg dithering [92]) can be used to produce a binary mask $\boldsymbol{c}\in \{0,1\}^{N}$ from a continuous density image $\boldsymbol{d}\in [0,1]^{N}$. The process involves iterating over the image pixels (e.g., in serpentine order), binarizing a single pixel at a given step, and then diffusing the error arising from the binarization to the set of currently nonvisited pixels. This results in a sequence of images $\boldsymbol{d}=\boldsymbol{d}^{1}, \boldsymbol{d}^{2},\ldots , \boldsymbol{d}^{N+1}=\boldsymbol{c}$. The binarization happens according to a thresholding step, which usually reads as

80 $$ c_{k} = d^{k+1}_{k} = \textstyle\begin{cases} 0 &\text{for } d^{k}_{k} < 0.5, \\ 1 &\text{for } d^{k}_{k} \geq 0.5. \end{cases} $$

Since we want to get multiple masks stochastically, we randomize the process by sampling a uniform random number $v_{k}\in [0,1]$ for pixel *k*, and then perform thresholding:

81 $$ c_{k} = d^{k+1}_{k} = \textstyle\begin{cases} 0 &\text{for } d^{k}_{k} < v_{k}, \\ 1 &\text{for } d^{k}_{k} \geq v_{k}. \end{cases} $$

Then the probability mass function for mask ***c*** constructed from density image ***d*** is

82 $$ p_{\boldsymbol{d}}(\boldsymbol{c}) = \frac{1}{P}\prod _{k=1}^{N} (d^{k}_{k}(\boldsymbol{c}))^{c_{k}} (1-d^{k}_{k}(\boldsymbol{c}))^{1-c_{k}}, \quad P = \sum _{\boldsymbol{c}\in \{0,1\}^{N}} \prod _{k=1}^{N} (d^{k}_{k}(\boldsymbol{c}))^{c_{k}}(1-d^{k}_{k}(\boldsymbol{c}))^{1-c_{k}}. $$

In the above $d^{k}_{k}(\boldsymbol{c})$ are assumed to be clamped to $[0,1]$. Note that while this bears similarity to Poisson sampling (see (64)), the probability $d^{k}_{k}(\boldsymbol{c})$ is conditioned on the probabilities in the *k* previous steps. Algorithmically, it is trivial to compute the numerator of the probability during the error diffusion process.

## Abbreviations

DbI: Denoising by Inpainting
DCT: Discrete Cosine Transform
EED: Edge-Enhancing Diffusion
MAP: Maximum A Posteriori
ML: Maximum Likelihood
MMSE: Minimum Mean Squared Error
MSE: Mean Squared Error
PDE: Partial Differential Equation
PDF: Probability Density Function
PMF: Probability Mass Function
RMSE: Root Mean Square Error
